# Why we thrive beneath a northern sky – genomic signals of selection in apple for adaptation to northern Sweden

**DOI:** 10.1038/s41437-024-00693-2

**Published:** 2024-06-04

**Authors:** J. Skytte af Sätra, L. Garkava-Gustavsson, P. K. Ingvarsson

**Affiliations:** 1 Department of Plant Breeding, Swedish University of Agricultural Sciences, Alnarp, Sweden; 2 Department of Plant Biology, Swedish University of Agricultural Sciences, Uppsala, Sweden

**Keywords:** Comparative genomics, Plant breeding

## Abstract

Good understanding of the genomic regions underlying adaptation of apple to boreal climates is needed to facilitate efficient breeding of locally adapted apple cultivars. Proper infrastructure for phenotyping and evaluation is essential for identification of traits responsible for adaptation, and dissection of their genetic composition. However, such infrastructure is costly and currently not available for the boreal zone of northern Sweden. Therefore, we used historical pomological data on climate adaptation of 59 apple cultivars and whole genome sequencing to identify genomic regions that have undergone historical selection among apple cultivars recommended for cultivation in northern Sweden. We found the apple collection to be composed of two ancestral groups that are largely concordant with the grouping into ‘hardy’ and ‘not hardy’ cultivars based on the pomological literature. Using a number of genome-wide scans for signals of selection, we obtained strong evidence of positive selection at a genomic region around 29 Mb_HFTH1_ of chromosome 1 among apple cultivars in the ‘hardy’ group. Using phased genotypic data from the 20 K apple Infinium® SNP array, we identified haplotypes associated with the two cultivar groups and traced transmission of these haplotypes through the pedigrees of some apple cultivars. This demonstrates that historical data from pomological literature can be analyzed by population genomic approaches as a step towards revealing the genomic control of a key property for a horticultural niche market. Such knowledge is needed to facilitate efficient breeding strategies for development of locally adapted apple cultivars in the future. The current study illustrates the response to a very strong selective pressure imposed on tree crops by climatic factors, and the importance of genetic research on this topic and feasibility of breeding efforts in the light of the ongoing climate change.

## Introduction

**Fig. 1:**
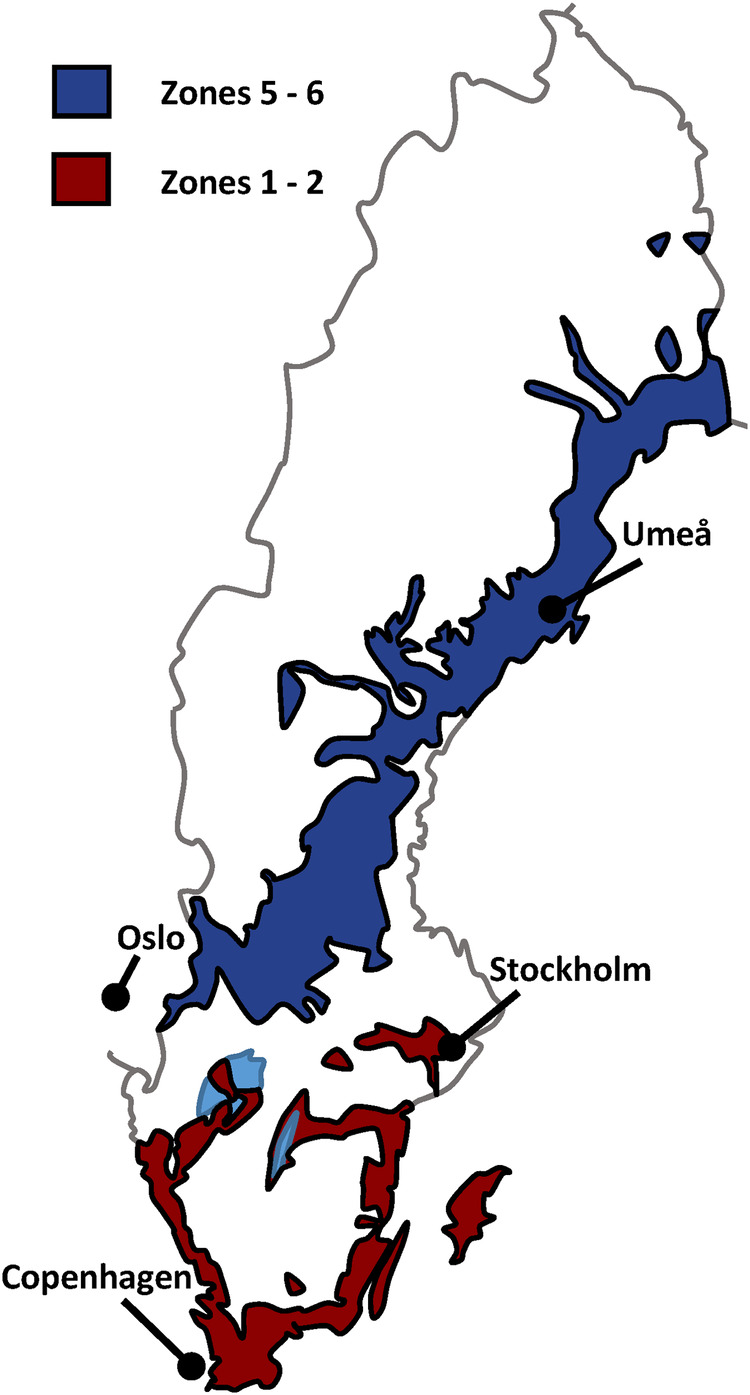
Simplified map of the Swedish horticultural climate zones, indicating zones 1–2 and zones 5–6. Modified from the Swedish Gardens zone map (Fernqvist 1993) with permission from the National Confederation of Swedish Gardens.

The domesticated apple (*Malus domestica* Borkh.) is one of the most widely cultivated fruit crops in the world. It is a diploid (2*n* = 2 x = 34) member of the Rosaceae family with life history traits typical of perennial fruit crops. It is a largely outcrossing species with a gametophytic self-incompatibility system and a long juvenile period. In addition, it is long-lived and often clonally propagated (Gaut et al. 2015). Consequently, a large proportion of genetic diversity is typically retained after domestication (Miller and Gross 2011). Sweden is located from 55°N to 69°N on the Scandinavian peninsula, with commercial apple production being concentrated to the southern part (55–57°N). However, some minor cultivation occurs around 59°N and commercial orchards, mainly for cider production, have recently been established around the city of Umeå at 63°N (Nybom 2019). Thus, there is a demand for novel apple cultivars adapted to northern Sweden. However, there is currently no infrastructure available for systematic evaluation of the performance of cultivars and selections in northern Sweden, although historical data are available for many existing cultivars, summarized in pomological literature (Näslund 2010; Nilsson 1987; Svensson 2005). Historical data on climate adaptation of horticultural lignified perennials in Sweden typically takes the form of recommended climate zones for cultivation, with the country divided into nine climate zones (1–8 and the alpine region) (Fernqvist 1993). The original system was published in 1910 and had only four climate zones, based only on meteorological observations, in particular the February isotherms. The system was subsequently revised based on empirical cultivation studies, and was eventually expanded to separate the current zones. In the present study, we considered apple cultivars recommended for zones up to 5–6 to be ‘hardy’, and apple cultivars recommended only for zones 1–2 to be ‘not hardy’ (Fig. 1). This largely corresponds to a division into apple cultivars adapted to the boreal zone and the continental zone of Europe, respectively (Metzger et al. 2005).

Adaptation to boreal climates in general, and northern Sweden in particular, has previously received little attention in apple and the impact of different candidate traits is yet to be validated in a systematic way. However, given the importance of the February isotherms for the definition of the climatic zones, freezing tolerance could be a strong candidate trait. In apple, studies based on the ectopic expression of a peach C-repeat binding factor (*CBF*) have shown that *CBF*’s and connected genes are important for the regulation of cold hardiness as well as growth, dormancy, and flowering (Wisniewski et al. 2015; Wisniewski et al. 2018). Abscisic acid (ABA) is a key regulator of cold stress tolerance in plants, partly acting through CBF-dependent pathways (Raza et al. 2023). Among deciduous trees in general, the timing of annual transitions between phenological stages is a key component of adaptation to boreal climate (Hänninen and Kramer 2007). In poplar, a model species for deciduous boreal trees, a region around a *PtFT2* locus has been found to have been very important for local adaptation (Ingvarsson and Bernhardsson 2020), affecting short-day induced growth cessation and bud set (Wang et al. 2018). In apple, studies of the genetic control of autumn phenology under Nordic conditions is still in its infancy (Skytte af Sätra et al. 2023a), though the genetic control of traits such as flowering (Urrestarazu et al. 2017; Allard et al. 2016) and fruit maturity date (Migicovsky et al. 2016; Migicovsky et al. 2021; Larsen et al. 2018; Minamikawa et al. 2021; Chagné et al. 2014) has received considerable attention internationally.

Recent years have opened up new possibilities for genetic and genomic studies in apple, due to dramatic advances in the availability of genotyping technologies, from simple-sequences repeats (SSRs) (Urrestarazu et al. 2016) to single nucleotide polymorphism (SNP) arrays (Bianco et al. 2016, 2014; Chagné et al. 2012), genotyping-by-sequencing (GBS) (Migicovsky et al. 2016), and whole genome re-sequencing (Duan et al. 2017). The differing characteristics of these technologies mean that they suit different applications, including population genetics and fingerprinting (SSRs) (Cornille et al. 2014; Denancé et al. 2020); pedigree reconstruction, QTL-mapping, and construction of dense linkage maps (SNP arrays) (Di Pierro et al. 2016; Howard et al. 2017; Rymenants et al. 2020); genome-wide association studies (GWAS, SNP arrays and GBS) (Larsen et al. 2019; Urrestarazu et al. 2017); and population genomic analyses (re-sequencing) (Chen et al. 2021). With the availability of high-quality reference genome sequences in apple (Daccord et al. 2017; Zhang et al. 2019), re-sequencing at high coverage offers unbiased high quality calls of SNPs and copy number variants (CNVs) at high density, allowing e.g., scans for signals of selection in a germplasm. However, sequencing at high coverage is relatively costly and downstream bioinformatics analyses are typically computationally intense. In contrast, genotyping with SNP arrays, in particular the Illumina Infinium® array, offers highly reproducible genotype calls at moderate densities and relatively low cost. It is therefore well suited for pedigree re-construction, which in turn allows for generation and curation of high-quality phased genotypic data (Vanderzande and Howard et al. 2019), facilitating e.g. tracing of founder haplotypes through apple pedigrees.

The aim of this study was to gain insights into the genomic basis for adaptation of apple to cultivation in the higher growing zones of Sweden. To achieve this, we coupled historical data on cultivation recommendations from pomological literature with high-coverage re-sequencing to scan for selective sweeps among apple cultivars considered ‘hardy’ in northern Sweden. We also identified haplotypes from the 20 K SNP array associated with hardiness in northern Sweden, or lack thereof, and traced them through the pedigrees of some relevant apple cultivars.

## Materials and methods

### Plant material and sequencing

The three most recent main Swedish pomologies were reviewed for historical data on cultivation zone recommendations (Näslund 2010; Nilsson 1987; Svensson 2005). Cultivars that were recommended for zone 5–6 were assigned as ‘hardy’ and cultivars that were recommended for zones 1–2 were assigned as ‘not hardy’. In total, 59 cultivars assigned to either of these groups were included in this study, based on their availability in apple gene banks hosted by the Swedish University of Agricultural Sciences (SLU). The two groups comprised 30 and 29 cultivars respectively, to obtain balanced population sizes. Cultivars of intermediate hardiness, recommended for zones 3–4, were not considered in this study. Leaf samples were collected from young growing shoots, freeze-dried at collection, and total genomic DNA was subsequently extracted using the DNeasy Plant Mini Kit (Qiagen) following the standard protocol. Malus UNiQue (MUNQ) genotype codes (Denancé et al. 2020) for each cultivar are given in Supplementary File S1, based on genotyping of the same sample as used in the current study or taken from the literature. MUNQs are also indicated throughout the manuscript for sequenced samples with verified MUNQs.

The DNA samples were sent to the SNP&SEQ Technology Platform at Uppsala University for library preparation and sequencing. Sequencing libraries were prepared from 1 μg DNA using the TruSeq PCRfree DNA sample preparation kit with an insert size of 350 base-pairs (bp). 150 bp paired-end sequencing was performed on a NovaSeq 6000 system, S4 flowcell, and v1.5 sequencing chemistry. The samples were sequenced to a theoretical depth of 17–39x per haploid genome (mean = 24).

### Mapping and SNP calling

Pair-end reads were aligned against the HFTH1v1 reference genome (Zhang et al. 2019) using the Maximal Exact Matches algorithm of the Burrows-Wheeler Aligner (BWA-MEM) (Li 2013) with default settings. Duplicates were marked and removed by MarkDuplicates of Picard (Broad Institute 2018). SNPs and insertion/deletions (INDELs) were called using HaplotypeCaller of GATK v4.2.0.0 (Poplin et al. 2018; Van der Auwera and O’Connor 2020).

Only SNPs mapped to contigs making up chromosome 1–17 of the HFTH1 reference genome were retained. SNPs were hard-filtered using BCFtools (Danecek et al. 2021) and VCFtools (Danecek et al. 2011) according to GATK best practices (Van der Auwera and O’Connor 2020), with parameters adjusted following inspection of the distributions of the parameters in the cohort (QD < 4.0; FS > 60.0; MQ < 40.0; MQRankSum < −6; MQRankSum > 5; ReadPosRankSum < −5.0; ReadPosRankSum > 5.0; SOR > 3.0).

Additional hard filtering of calls was performed as recommended by Bernhardsson et al. (2020). Individual SNP calls were filtered such that calls with depth below 6 or above 44, or with GQ below 17, were called as missing. SNP loci were then filtered across the cohort, excluding loci with an average depth below 10, above 32, more than 20% missing values, and those with more than two alleles. Finally, loci with *p*-values for excess heterozygosity below 0.05 were removed. In addition, loci with extreme outlier values for variance in read depth (VAR_DEPTH > 500) were excluded, in order to mitigate potential allelic dropouts (O’Leary et al. 2008).

### Analysis

#### Genetic structure and LD decay

Genetic structure was assessed for one to five subpopulations (K) using ADMIXTURE v.1.3.0 (Alexander et al. 2009), with five-fold cross-validation using a subset of 267,963 loci pruned for local linkage disequilibrium (LD) (window 50 loci, *r*^*2*^ = 0.1) with minor allele frequency (maf) > 0.05 and max 10% missing data.

Principal component analysis (PCA) was performed using single nucleotide polymorphism (SNP) loci after LD pruning (window 100 loci, *r*^*2*^ = 0.2, leaving 256,184 SNP’s with maf > 0.05 and less than 10% missing data), using PLINK v1.9 (Chang et al. 2015; Purcell et al. 2007). Pairwise kinship coefficients were estimated using KING (Manichaikul et al. 2010).

**Fig. 2:**
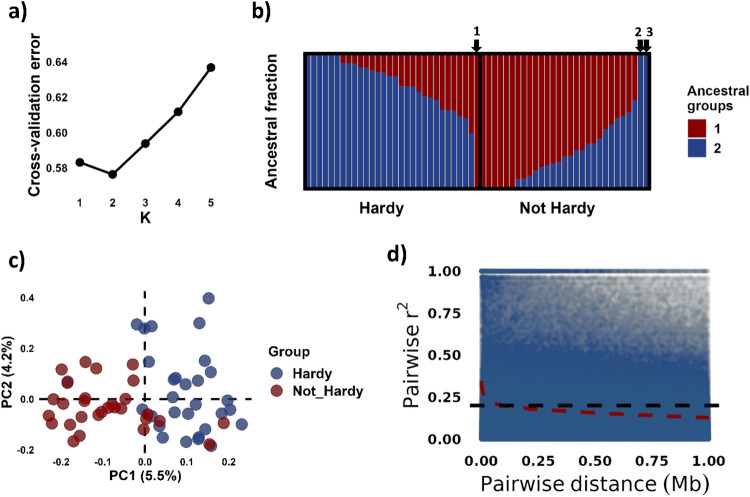
Genetic structure and LD decay for the studied germplasm. **a** Cross-validation errors for different numbers of ancestral groups, **b** ancestral fractions of the cultivars by the groups ‘hardy’ and ‘not hardy’ for K = 2, **c** principal component analysis (PCA) plot, and **d** linkage disequilibrium (LD) decay across the cohort. In **d**, a subset of 1 M randomly selected pairwise comparisons are plotted, while the trend line (red, dashed) is based on the full dataset. The arrows indicate the ancestral fractions of (1) ‘Släthultsäpple’, (2) ‘Leckö Astrakan’, and (3) ‘Noors Glasäpple’.

Chromosome-wide LD decay was estimated by pairwise *r*^*2*^ and was calculated within each chromosome using PLINK v1.9 (Chang et al. 2015; Purcell et al. 2007), considering only pairs of loci less than 1 Mb apart. A randomly chosen subset of loci with maf > 0.05 and max 10% missing data was used, with each locus having a probability of 0.1 of inclusion. LD decay was calculated in sliding windows of pairwise distances in bins of 1 kb (Fig. 2) or 20 bp (Supplementary File S2), as median and average over all chromosomes. LD decay was estimated for the entire dataset, and for the ‘hardy’ and ‘not hardy’ groups separately, excluding the three cultivars with major ancestral group fractions conflicting with the group assignment (‘Släthultsäpple’ [MUNQ 10826], ‘Leckö Astrakan’ [MUNQ 10806], and ‘Noors Glasäpple’ [MUNQ 10816]).

### Scans for selective sweeps

Composite likelihood ratio (CLR) tests for positive selection were performed separately for the ‘hardy’ and ‘not hardy’ groups (Supplementary File S2) using SweepFinder2 (Degiorgio et al. 2016; Nielsen et al. 2005), with 1 kb between grid points.

For calculations of cross-population extended haplotype homozygosity (XP-EHH), all loci with missing values were excluded, leaving 9.6 M SNPs. XP-EHH calculations were performed with selscan v2.0 (Sabeti et al. 2007; Szpiech 2022; Szpiech and Hernandez 2014) using unphased data, including loci at the chromosome boundaries, and utilizing physical (HFTH1) map positions. XP-EHH values were standardized in frequency bins using norm, with default settings.

Pairwise F_ST_, nucleotide diversity (π), and Tajima’s D were calculated using VCFtools (Danecek et al. 2011), in windows of 10 kb. A case/control genome-wide association study was performed using PLINK v.1.9 (Chang et al. 2015; Purcell et al. 2007), with individuals in the ‘hardy’ group as the ‘case’ and individuals in the ‘not hardy’ group as the ‘control’. Local LD structure in the candidate region around the selective sweep on chromosome 1 was calculated in PLINK v1.9 (Chang et al. 2015; Purcell et al. 2007) for the ‘hardy’ and ‘not hardy’ groups separately, using all SNPs.

### 20K SNP array data

Genotypic data from the 20 K apple Infinium® SNP array (Bianco et al. 2014) were available for a wide Nordic germplasm from previous and ongoing research projects (Skytte af Sätra 2023; Skytte af Sätra et al. 2020), or kindly made available through Eric van de Weg and Michela Troggio on behalf of FruitBreedomics. Marker data were curated and phased as described by Vanderzande and Howard et al. (2019). In this study, only markers mapped to linkage group (LG) 1 on the iGLMap (Di Pierro et al. 2016) and retained in a study on marker integration (Howard et al. 2021) were considered, in order to trace the inheritance of the locus showing signals of selection on chromosome 1 in the ‘hardy’ group. SNPs were called in Genome Studio (GS) v 2.0 (Illumina Inc.) starting from cluster definitions kindly made available by Howard et al. (2021). Genetic and HFTH1 positions for the SNP markers were taken from an advanced draft of the 15K-iGW-map (Skytte af Sätra et al. 2023b). To get more informative haplotype information for the selected locus around 50cM_iGW_ we considered SNPs that might have been mapped on the iGLMap but discarded from the study on marker integration (Howard et al. 2021). On doing that, we found two additional SNP markers on the 15K-iGW-map (index 9357 and 2274) to complement the target locus. Note that SNP index 9357 exhibited variation in signal intensity across the analyzed germplasm, due to one or several polymorphisms or INDELs in the region targeted for probe hybridization (Di Guardo et al. 2015; Vanderzande et al. 2019). Thus, a *null* allele was called at this locus in addition to the *T* and *C* alleles of the polymorphism targeted by the probe design. Identity-by-Decent (IBD) probabilities were calculated for haploblock marker data in FlexQTL™ (www.flexqtl.nl) and visualized in Pedimap (Voorrips et al. 2012).

## Results

### Sequencing, structure, and LD decay

A total of 59 apple cultivars selected for re-sequencing based on a review of pomological literature (Supplementary File S1) were sequenced to an average depth of ~24x of haploid apple genome. The final dataset contained 17.3 M biallelic SNPs after quality filtering. The assessments of estimated ancestry revealed the ‘K = 2’ model to have the lowest cross-validation error, indicating that it is a sensible modeling option for the current dataset (Fig. 2a). Considering the ‘K = 2’ model, the ancestral fractions were significantly different between the ‘hardy’ and ‘not hardy’ groups (*p* « 0.001) (Fig. 2b). The ‘hardy’ group had mean ancestral fraction of 0.77 for ancestral group ‘2’, while the ‘not hardy’ group had a mean ancestral fraction of 0.71 for ancestral group ‘1’. Accordingly, the PCA revealed two weakly defined groups separated along PC1, largely corresponding to the ‘hardy’ and ‘not hardy’ groups (Fig. 2c). The two first PCs explained 9.7% of the genetic variance in the dataset, after pruning of SNPs in LD. The inferred kinship coefficients exhibited similar distributions within the two respective groups and between groups, with mean values of −0.02, −0.03, and −0.05 for ‘hardy’-‘hardy’, ‘not hardy’-‘not hardy’, and cross-group comparisons, respectively (Supplementary File S2).

Notably, three cultivars deviated from this general pattern. The cultivar ‘Släthultsäpple’ [MUNQ 10826] assigned to the ‘hardy’ group had an ancestral fraction of 1.0 for ancestral group ‘1’ and clustered together with the cultivars in the ‘not hardy’ group in the PCA plot. In contrast, the cultivars ‘Leckö Astrakan’ [MUNQ 10806] and ‘Noors Glasäpple’ [MUNQ 10816] had an ancestral fraction of 1.0 for ancestral group ‘2’ and clustered together with the cultivars in the ‘hardy’ group in the PCA plot. However, as there were no indications in our previous work (Skytte af Sätra et al. 2020) that there are issues with true-to-typeness of these samples, they were retained for downstream analysis.

Across the entire dataset, the average chromosome-wide LD was found to decay to *r*^*2*^ below 0.2 after 93 kb, averaged over all chromosomes (Fig. 2d). Considering the two groups separately, local LD was found to decay even more slowly, dropping below 0.2 around 200 kb (Supplementary File S2). However, as the distribution of pairwise *r*^*2*^ was highly skewed, the median might be a more representative estimate of LD decay. In the full dataset, the median *r*^*2*^ for the 0–20 bp bin was already 0.19 (Supplementary File S2).

### Genome-wide scans for signals of selection

**Fig. 3:**
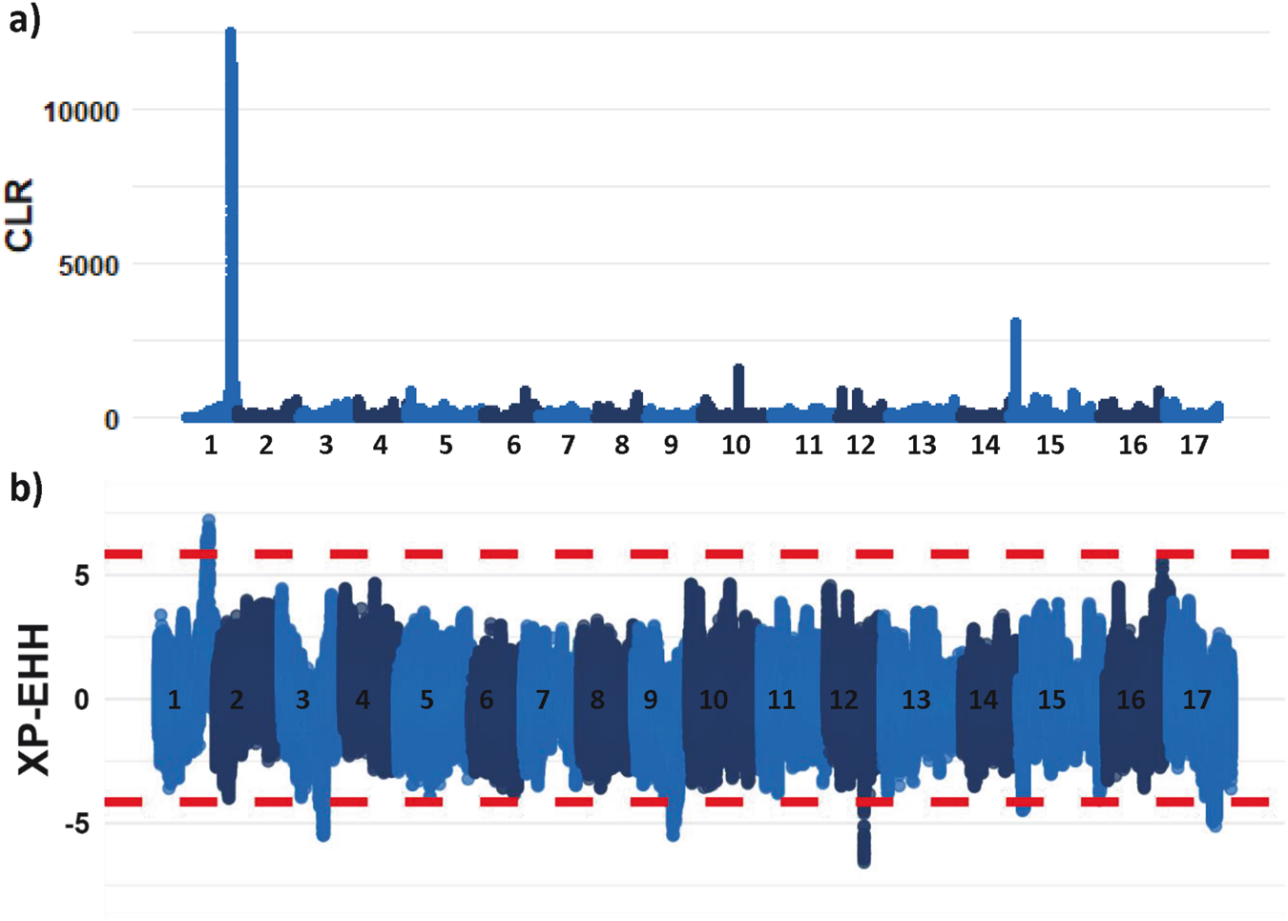
Genome-wide scans for signals of selection. Genome-wide Manhattan plots of **a** composite likelihood ratio (CLR) in the ‘hardy’ group and **b** standardized extended haplotype homozygosity (XP-EHH) between groups, with upper and lower boundaries for 0.1% outliers indicated by dashed red lines. Chromosome numbers (1–17) are indicated along the x-axes.

A distinct peak with high CLR indicated presence of a selective sweep on chromosome 1 that was restricted to the ‘hardy’ group (Fig. 3a). In the ‘not hardy’ group, the highest peak identified by the CLR test was observed at the top of chromosome 15 (Supplementary File S2), coinciding with a minor peak that was present also in the ‘hardy’ group. Similarly, a distinct positive peak in XP-EHH was also found on chromosome 1 (Fig. 3b), again indicating a selective sweep that occurred in the ‘hardy’ group. Five negative peaks in XP-EHH below the 0.1% outlier threshold were also seen (chromosomes 3, 9, 12, 15, and 17; Fig. 3b), indicating positive selection in the ‘not hardy’ group.

### Characterization of the locus on chromosome 1

**Fig. 4:**
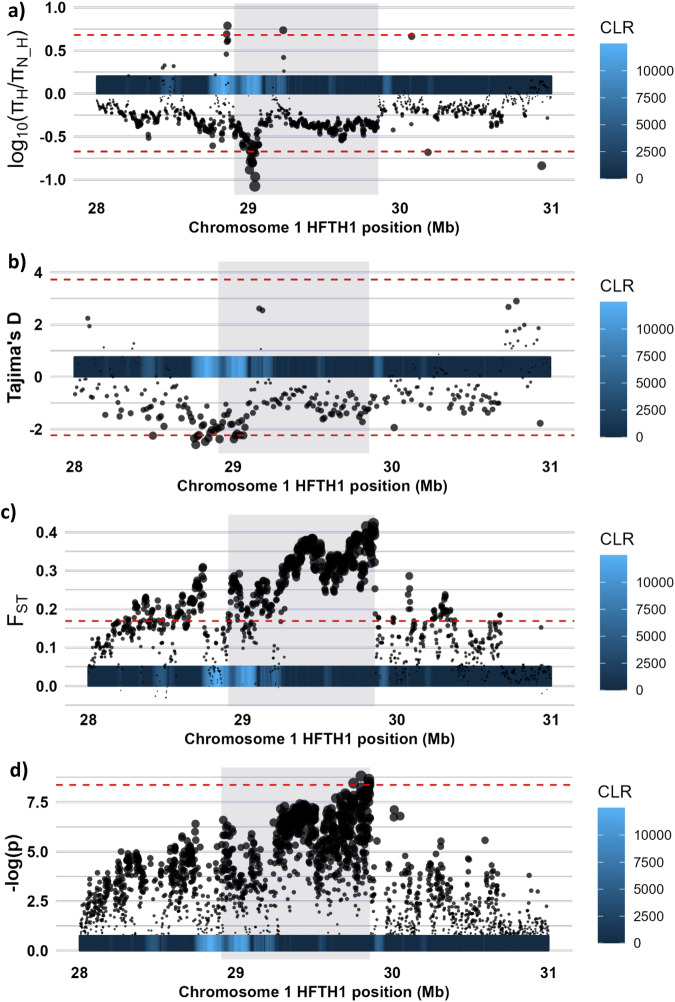
Close-up view of the 28–31 Mb region of chromosome 1. Illustrating (**a**) log_10_-transformed scaled diversity, i.e., log_10_-transformed ratio of π in the ‘hardy’ and ‘not hardy’ groups, **b** Tajima’s D for the ‘hardy’ group, **c** weighted pairwise F_ST_, and **d** -log_10_-transformed *p*-values from a case/control GWAS in averaged in 1 kb sliding windows. Dashed red lines indicate genome-wide 0.1% outlier thresholds (a-c) and threshold for *p* < 0.05 following genome-wide Bonferroni correction of individual SNP loci (**d**). The region with XP-EHH above the genome-wide 0.1% outlier threshold is indicated by a light-blue background, and the local CLR values for the ‘hardy’ group are given as a heatmap bar along the x-axis of each panel.

As both the CLR test for the ‘hardy’ group and the assessment for XP-EHH provided indications of a selective sweep on the distal part of chromosome 1 in the ‘hardy’ group, we further characterized the patterns in genetic diversity in this region. Assessments of scaled diversity (π_Hardy_/π_Not Hardy_) showed a clear drop around 29.0 Mb, indicating a reduction in genetic diversity of 10% in the ‘hardy’ group relative to the ‘not hardy’ group. In contrast, the genome-wide average for the scaled diversity was close to zero (mean log_10_[π_Hardy_/π_Not Hardy_] = 0.0020), indicating that the two groups had very similar levels of genetic diversity. Local assessments of Tajima’s D dropped below the genome-wide 0.1% outlier threshold around 28.8 Mb in the ‘hardy’ group, indicating an excess of rare alleles in this region. In accordance with a selective sweep around 29 Mb in the ‘hardy’ group, the flanking regions exhibited increased values in pairwise F_ST_, skewed towards 29.9 Mb, with a drop around 29 Mb coinciding with the drop in scaled diversity. Lastly, a case/control GWAS for assignment to the ‘hardy’ and ‘not hardy’ group indicated a single peak on chromosome 1 from several loci with –log(p) values exceeding the threshold for genome-wide Bonferroni correction. Similar to the peak in F_ST_, the GWAS peak was skewed towards 29.9 Mb, rather than the 29 Mb region suggested by the scaled diversity and Tajima’s D. As expected from the peak in XP-EHH, the ‘hardy’ group had a stronger LD structure around the center of the selective sweep on chromosome 1 (28.9–29.1 Mb_HFTH1_) with an average *r*^*2*^ of 0.24, compared to an average *r*^*2*^ of 0.18 in the ‘not hardy’ group. The ‘hardy’ group also had an LD-block at approximately 29.00–29.05 Mb_HFTH1_ with and average *r*^*2*^ of 0.35, while the ‘not hardy’ group had an average *r*^*2*^ of 0.27 in the same region (Supplementary File S2). Thus, there is a preponderance of evidence for positive selection acting on the ‘hardy’ cultivars targeting a region located around 29 Mb of chromosome 1 (Fig. 4, Supplementary File S2).

### 20K SNP array haplotypes

Phased genotypic data from the 20 K SNP array were available for 21 individuals from the ‘hardy’ group and 16 individuals from the ‘not hardy’ group. The SNPs on the 20 K array are designed to form focal points along the apple genome, and a cluster of five SNP markers spanning less than 0.5 cM_iGW-map_ (28.9–29.1 Mb_HFTH1_) targeted the center of the selective sweep detected in the ‘hardy’ group. Among these 37 individuals, the alleles of the five SNPs formed five haplotypes, present at different frequencies in the two groups. Only three of the haplotypes were present in the ‘hardy’ group, indicating lower genetic diversity in the region, as expected under a selective sweep. One of the haplotypes (#1) had a frequency above 0.8 in the ‘hardy’ group and was more than five times as common in the ‘hardy’ group as in the ‘not hardy’ group. In contrast, all five haplotypes were present in the ‘not hardy’ group, with haplotype (#2) having the highest frequency in this group. Notably, this haplotype was completely absent in the ‘hardy’ group (Table 1).

**Table 1:** Haplotypes in the ‘hardy’ and ‘not hardy’ groups, based on genotypic 20 K SNP array data, phased through pedigree connections.

HT #	20 K array SNP index					F(*Hardy*)	F(*Not Hardy*)	F_*H*_ / F_*N H*_
	2274	9357	9358	9359	9360			
1	*A*	*null*	*C*	*A*	*T*	0.86	0.16	5.5
2	*G*	*C*	*T*	*A*	*G*	–	0.47	–
3	*A*	*T*	*C*	*A*	*G*	0.10	0.22	0.4
4	*A*	*null*	*C*	*A*	*G*	0.05	0.09	0.5
5	*A*	*null*	*C*	*G*	*G*	–	0.06	–

For each haplotype (numbered 1–5), the calls for each of the five SNPs are given, as well as the frequency of the haplotype in the ‘hardy’ (F(*Hardy*)) and the ‘not hardy’ group (F(*Not Hardy*)), respectively, as well as the relative frequency in the ‘hardy’ group relative to the ‘not hardy’ group (F_*H*_/F_*N H*_).

**Fig. 5:**
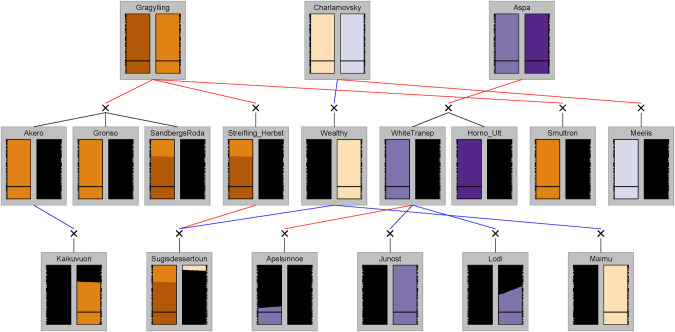
Transmission of LG1 haplotypes through some descendants of three cultivars (‘Grågylling’, ‘Charlamovsky’, and ‘Aspa’) in the ‘hardy’ group that are all homozygous for haplotype #1 (Table 1). Each vertical bar represents one homolog with genetic positions according to the 15K-iGW-map, and the position of the selective sweep in the ‘hardy’ group is indicated by a black horizontal bar at 50 cM_iGW-map_. Haplotype segments from other founders are colored black.

**Fig. 6:**
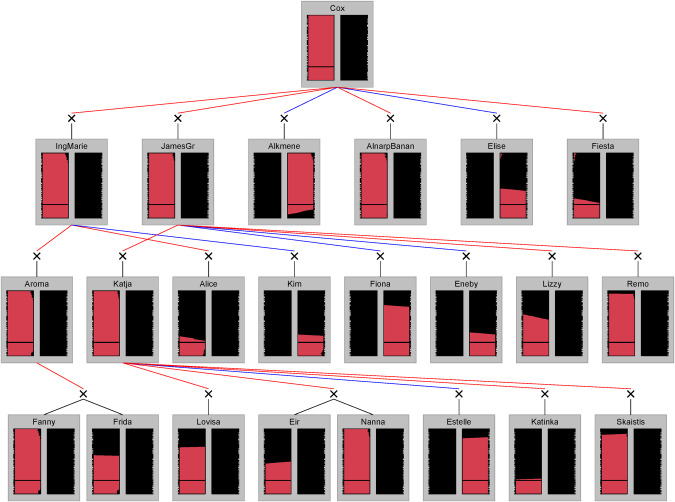
Transmission of the LG1 haplotype of ‘Cox’s Orange Pippin’ from the ‘not hardy’ group carrying haplotype #2 (Table 1) to some descendants. Each vertical bar represent one homolog, and the position of the selective sweep in the ‘hardy’ group is indicated by a black horizontal bar at 50 cM_iGW-map_. Haplotype segments from other founders are colored black.

We were also able to trace the 20 K SNP haplotypes through verified and reconstructed pedigrees of some apple cultivars (Muranty et al. 2020; Skytte af Sätra 2023; Skytte af Sätra et al. 2020). This analysis revealed that many founders of the ‘hardy’ group were homozygous for a specific identical-by-state (IBS) haplotype (HT #1, Table 1). All cultivars in the ‘hardy’ group which are known founders or semi-founders in the pedigree (‘Aspa’ [MUNQ 1855], ‘Charlamovsky’ [MUNQ 29], ‘Grågylling’ [MUNQ 1806], ‘Melba’, and ‘Stenbock’ [MUNQ 5518]) were found to be homozygous for haplotype #1. Additionally, two cultivars previously identified as key founders among the Swedish heirloom cultivars (‘Gimmersta’ and ‘Vitgylling’) (Skytte af Sätra et al. 2020) were both homozygous for haplotype #1 (Fig. 5). Considering the haplotypes that were unique to the ‘not hardy’ group, ‘Cox’s Orange Pippin’ [MUNQ 163] carried one copy of haplotype #2. ‘Cox’s Orange Pippin’ is a key founder for Swedish apple production, as its descendants ‘Ingrid-Marie’ (offspring), ‘Aroma’ (grand-offspring), and ‘Frida’ (great-grand-offspring) together constitute more than half of Sweden’s commercially planted apple trees (SJV, 2017). All three of these descendants have inherited haplotype #2 from ‘Cox’s Orange Pippin’ (Fig. 6) and none of them is considered ‘hardy’ in northern Sweden. The two cultivars ‘Elise’ and ‘Katja’ (Eng. ‘Katy’), which represent a smaller share of commercially planted apple trees in Sweden (together < 7%), but which have also inherited haplotype #2 from ‘Cox’s Orange Pippin’, are also not considered ‘hardy’ in northern Sweden. Of the five key founders among Swedish heirloom cultivars previously identified (Skytte af Sätra et al. 2020), ‘Klockhammarsäpple’ was the only one not homozygous for haplotype #1, being heterozygous for haplotypes #1 and #4.

## Discussion

### Genetic structure and LD

The 59 cultivars sequenced in this study were assigned to two different ancestral groups and formed two weakly separated clusters along the first PC. This is in line with findings in previous studies indicating that the European apple germplasm consists of three subgroups. These three subgroups were identified as ‘Southern Europe’, ‘Western Europe’, and ‘North-Eastern Europe’, consistent with the geographical regions of origin of the cultivars (Urrestarazu et al. 2016). Of the samples included in that study obtained from the gene bank at the Swedish University of Agricultural Sciences, the vast majority were assigned to the ‘Western Europe’ and the ‘North-Eastern Europe’ groups, and only a very minor fraction to the ‘Southern Europe’ group. The ancestral groups identified in the present study seem to be largely in line with the cultivars of Urrestarazu et al. (2016), with e.g., ‘Cox’s Orange Pippin’ [MUNQ 163], ‘Beauty of Bath’, ‘Discovery’, and ‘King of the Pippins’ [MUNQ 37] in Urrestarazu et al.’s (2016) ‘Western Europe’ subgroup being assigned to ancestral group 1. Similarly, cultivars like ‘Suislepp’ [MUNQ 239], ‘Charlamovsky’ [MUNQ 29], and ‘Antonovka Obyknovennaja’ in the ‘North-Eastern Europe’ of Urrestarazu et al. (2016) had a major proportion of ancestry to ancestral group 2. Similar patterns of gene flow from Eastern Europe to Sweden have been reported in e.g., the boreal tree species European aspen (*Populus tremula* L.) (Rendón-Anaya et al. 2021). The assignment to the groups ‘hardy’ and ‘not hardy’ was mostly consistent with the major ancestral group of the cultivars and their clustering along the first PC. However, there were three clear outliers. The cultivar ‘Släthultsäpple’ [MUNQ 10826] in the ‘hardy’ group had an ancestral fraction close to one for ancestral group 1 and clustered with the ‘not hardy’ cultivars, close to ‘Pricilla’, on the PCA plot. The two cultivars likely share a common ancestor, with ‘Winesap’ being a parent of ‘Släthultsäpple’ and grandparent of ‘Priscilla’ through ‘Delicious’, although the two cultivars are otherwise unrelated. On the other hand, ‘Noors Glasäpple’ and ‘Leckö Astrakan’ were assigned to the ‘not hardy’ group, but had ancestral fractions close to one for ancestral group 2 and clustered with the ‘hardy’ group on the PCA plot. Both cultivars are offspring of two key founders among Swedish heirloom cultivars (Skytte af Sätra et al. 2020), ‘Gimmersta’ and ‘Vitgylling’. Both ‘Gimmersta’ and ‘Vitgylling’ are homozygous for haplotype #1 at the locus undergoing selection among cultivars in the ‘hardy’ group. However, ‘Vitgylling’ is only recommended for cultivation up to zone 4, while for ‘Gimmersta’ no recommendations are available in the pomological literature. It is important to note that historical data such as those used for grouping cultivars in this study hold several possible sources of error. One obvious issue is that the genotype evaluated under a given name might not be the genotype referred to by the same name in the clonal archives. Moreover, there could be historical bias in the cultivars evaluated, e.g., for northern Sweden, such that some cultivars were recommended only for southern Sweden simply because they were never evaluated in the north. Nevertheless, in the present study we found clear signals of strong positive selection at a clearly defined region. In addition, we observed signals of selection in line with a previously reported domestication locus for apple (Chen et al. 2021; Liao et al. 2021).

In the full dataset in the present study, we found average pairwise *r*^*2*^ to decay below 0.2 after 93 kb and to 0.16 after 500 kb, which is relatively long for an outcrossing species. Previous work has reported varying levels of LD decay in apple for different germplasms, e.g., Urrestarazu et al. (2017) reported average pairwise *r*^*2*^ to have decayed below 0.2 after 5 kb.

On the other hand, Vanderzande et al. (2017) studied germplasm from Belgium and found average pairwise *r*^*2*^ decay to below 0.2 only after 100 kb and to 0.16 after 500 kb, while also observing a significant genetic structure with the germplasm comprising two subgroups. These estimates are clearly in line with those in our study. National gene bank collections generally tend to contain accessions with extensive pedigree connections (Larsen et al. 2018; Skytte af Sätra et al. 2020; Vanderzande et al. 2017), which increases LD in the germplasm. Several verified close pedigree connections exist among the accessions in the present study, mainly among cultivars within the ‘hardy’ and ‘not hardy’ groups, respectively (Supplementary Files S1 and S2). This is probably one reason for the unexpectedly long range of LD observed, as evidenced by LD decaying even more slowly when estimated for the two groups separately, decaying to 0.2 only after approximately 200 kb. On the other hand, most individuals appear to be unrelated, both within and between groups (Supplementary File S2), though the observed population structure lead to increased LD (Fig. 2, Supplementary File S2). Thus, another reason for slow LD decay might be the dense SNP data in combination with a small cohort, resulting in a high frequency of outlier pairs with an *r*^*2*^ of one, causing a skew in the mean *r*^*2*^. Considering the skew of the distribution of pairwise *r*^*2*^ values, the median might be a more appropriate estimate of LD decay rates than the average. We found the median *r*^*2*^ to be below 0.2 for pairwise comparisons of loci only 1–20 bp apart (Supplementary File S2).

### Selective sweeps on chromosomes 1 and 15 among ‘hardy’ apple cultivars

Sweden, situated on the Scandinavian peninsula of northern Europe, hosts a wide range of climate regions. Southern-coastal parts, where a majority of Sweden’s commercial apple production is located, belong to the continental zone, similar to eastern Germany and Poland. On the other hand, the northern part belongs to the boreal, or even alpine north, environmental zone, with the nemoral zone in between (Metzger et al. 2005). Thus, apple cultivars suitable for cultivation in northern Sweden need to be adapted to a climate similar to that of Finland, western Russia, and inland parts of the Baltic countries. This climate differs greatly from that in the major apple-producing regions of Europe. Our scans for signals of selection revealed a distinct peak around 29 Mb_HFTH1_ of chromosome 1 in apple cultivars recommended for cultivation in northern Swedish climate zones (zones 5–6). Similar studies in European aspen have also identified a single locus exhibiting strong signals of positive selection in populations from northern Scandinavia (Rendón-Anaya et al. 2021). In addition, we found a minor CLR peak around 3.5 Mb_HFTH1_ of chromosome 15. Similarly, we observed a much stronger peak around 3.0 Mb_HFTH1_ of chromosome 15 among the apple cultivars recommended for cultivation only in the southern-most parts of Sweden (zones 1–2). This is in line with previous findings that a homolog of the *Arabidopsis ERECTA* gene was an important domestication locus associated with fruit size in apple, located around 3.4 Mb_GDDH13_ of chromosome 15 (Chen et al. 2021; Liao et al. 2021). Thus, our results are consistent with previous findings, to the extent that such is available. However, further attempts at pinpointing the site under selection using the current data will likely prove futile, due to the inconsistencies between metrics (Fig. 3), the relatively wide region showing signals of selection as is typical of a strong sweep, and the fact that it is not uncommon for sweeps to be asymmetrical around sites undergoing selection (Walsh and Lynch 2018). Nevertheless, even with a relatively large genomic region, this information can be used as basis for further work on the genetic mechanisms behind adaptation to northern Sweden, and development of tools for marker-assisted breeding for this property.

### Transmission of founder haplotypes

Using phased genotypic data from the 20 K SNP array in a pedigreed germplasm (Skytte af Sätra 2023; Skytte af Sätra et al. 2020; Vanderzande and Howard et al. 2019), we identified haplotypes co-localizing with the selective sweep on chromosome 1 that were over-represented among the cultivars recommended for cultivation in northern Sweden. Conversely, we identified haplotypes that were found solely among cultivars only recommended for cultivation in the southern-most parts of Sweden. Interestingly, one of the most important founders for modern Swedish apple cultivars, ‘Cox’s Orange Pippin’ [MUNQ 163], is recommended for cultivation only in southern-most Sweden and carries one copy of a haplotype that appears to be associated with this property in the germplasm. Accordingly, ‘Cox’s Orange Pippin’ clustered with group 1, with an ancestral fraction of 1.0, which is consistent with the ‘not hardy’ group. The French cultivar ‘Margil’, commonly used for cider production, has previously been identified as one of the parents of ‘Cox’s Orange Pippin’. A very large number of popular European apple cultivars are descendants of ‘Margil’, although it has been found to have a very weak genetic influence on apple cultivars suitable for northern Sweden (Muranty et al. 2020; Nybom 2020). Interestingly, the haplotype of ‘Cox’s Orange Pippin’ at the chromosome 1 locus that seems to be associated with a lack of adaptation to northern Sweden appears to originate from ‘Margil’ (Howard et al. 2018). We found that this haplotype has been transmitted to many of the most popular apple cultivars for commercial production in southern Sweden. This is partly in line with our previous observation that available genetic resources of heirloom cultivars which are ‘hardy’ for cultivation in northern Sweden have been underutilized in modern Swedish apple breeding (Skytte af Sätra et al. 2020). It has also been noted that ‘Klockhammarsäpple’ has a relatively low genetic contribution to cultivars recommended for Swedish climatic zones > 2 compared with the other four key founders among Swedish heirloom cultivars (‘Gimmersta’, ‘Grågylling’ [MUNQ 1806], ‘Vitgylling’, and ‘Rosenhäger’). ‘Klockhammarsäpple’ is also the cultivar with the least offspring among Swedish heirloom cultivars, among the five key founders (Nybom and Skytte af Sätra 2021; Skytte af Sätra et al. 2020). Given the findings in the present study, this could in part be because ‘Klockhammarsäpple’ is the only one of the five that is not homozygous for the haplotype at chromosome 1 associated with adaptation to northern Sweden. While the information on 20 K haplotypes should be considered provisional until their correspondence with phased genome sequences has been confirmed, it could be used to design crosses for further studies of adaptation to northern Sweden.

### Candidate genes around the chromosome 1 locus

In accordance with a strong selective sweep, the region showing signals of selection on chromosome 1 was relatively wide. Interestingly, this region did not appear to overlap with previously reported locations for speculated candidate loci, e.g. *MdCBF*1, 2, and 4 (chromosome 7, 6, and 4; Wisniewski et al. 2015), flowering (Allard et al. 2016; Urrestarazu et al. 2017), and fruit maturity date (Migicovsky et al. 2016; Migicovsky et al. 2021; Larsen et al. 2018; Minamikawa et al. 2021; Chagné et al. 2014).

The center of the selective sweep on chromosome 1 (28.9–29.9 Mb_HFTH1_) co-localized with 18 predicted genes of the HFTH1 WGS v1.0 according to the Genome Database for Rosaceae (GDR; www.rosaceae.org; Jung et al. 2018 [accessed 230817]). Eight of these represents a cluster of genes with transcript similarity to cytochrome P450 of *Arabidopsis* (HF23674, HF23673, HF23672, HF23671, HF23670, HF23669, HF23668, HF23666; 28,965,818–29,069,022 nt_HFTH1_). Cytochrome P450 enzymes are involved in a wide range of stress responses in plants, although P450s can also be involved in regulation of growth-related phytohormones (Xu et al. 2015). Interestingly, CYP707A degrades ABA and has been suggested to be an important regulator of endodormancy as its expression levels increased during chilling accumulation in both pear and peach (Pan et al. 2021; Wang et al. 2016; Li et al. 2018), while in *Arabidopsis* it appears to be involved in regulation of seed dormancy. Seven of the P450 genes in the center of the selective sweep have transcripts with the highest similarity to CYP704A of *Arabidopsis*. While little is currently known about the specific functions of CYP704A in plants, CYP704A2 has the highest expression in dry seeds in *Arabidopsis* according to the *Arabidopsis* Information Resource (TAIR; www.arabidopsis.org; Berardini et al. 2015). In maize and rice, expression of CYP704A transcripts have been found to increase during drought stress (Li and Wei 2020). If any of these P450 genes, or the entire cluster, has indeed been the actual target for the observed selective sweep on chromosome 1, this might indicate that an important factor in boreal climate adaptation in apple shares networks with seed processes of *Arabidopsis*, similar to how the regulatory networks for floral initiation in *Arabidopsis* is similar to that for vegetative growth in *Populus* sp. (Ding and Nilsson 2016).

## Conclusions

Adaptation to a specific climate is a complex property that may depend on several traits, and solid scientific knowledge about those traits may not always be available. By combining whole genome resequencing with historical data from pomological literature, we found a preponderance of evidence for a selective sweep on chromosome 1 among cultivars recommended for cultivation in northern Sweden. Using phased genotypic data from the 20 K SNP array for a collection of pedigreed apple cultivars we identified a haplotype comprising five SNPs associated with adaptation to northern Sweden, and one haplotype associated with lack of adaptation to northern Sweden. While further work is needed to identify the traits associated with this region and validate their effect, this study represents the first step towards understanding the genomic basis for adaptation of cultivated apple to northern Sweden. The very clear signal for positive selection towards boreal adaptation implies a strong selective pressure, which illustrates the importance of climate adaptation in apple as an example of a horticultural tree crop. This study paves the way for further studies on climate adaptation in other tree crops, in e.g. horticulture and forestry. As the global climate is currently changing rapidly, this stresses the need for more genetic research and targeted breeding efforts to ensure a sustainable development in regional and global food production.

### Supplementary information

Supplementary File 1

Supplementary File 2

## Data Availability

The whole genome sequencing raw reads have been deposited in ENA, project accession PRJEB71391. Genotype calls from the 20 K SNP array are part of other ongoing research projects; calls of the sequenced individuals can be made available for LG1 upon request.
