# Supplementary material for: Why we thrive beneath a northern sky – genomic signals of selection in apple for adaptation to northern Sweden
Source: Heredity (Edinb). 2024 Jun 4;133(2):67–77. doi: 10.1038/s41437-024-00693-2 (PMC11286948; doi:10.1038/s41437-024-00693-2)
Supplement: Supplementary file 2 [file 41437_2024_693_MOESM2_ESM.docx]

**Supplemental File 2**

Skytte af Sätra J^1^*, Garkava-Gustavsson L^1^ and Ingvarsson PK^2^ (2024) *Why we thrive beneath a northern sky – Genomic signals of selection in apple for adaptation to northern Sweden*, Heredity

^1^Department of Plant Breeding, Swedish University of Agricultural Sciences, Alnarp, Sweden; ^2^Department of Plant Biology, Swedish University of Agricultural Sciences, Uppsala, Sweden

*Corresponding author: jonas.skytte.af.satra@slu.se

Decay of linkage disequilibrium (LD) was calculated for the Hardy and the Not Hardy groups separately, excluding ‘Släthultsäpple’, ‘Leckö Astrakan’, and ‘Noors Glasäpple’ (Fig. 1 and 2). Differences between average R^2^ assessments and median R^2^ assessments are shown in figure 3. Distributions of R^2^ values over different ranges of pairwise distances are shown in figures 4 to 9. Distributions of pair-wise kinship coefficients are given in figure 10.

Genome-wide composite likelihood-ratio (CLR) tests in the not hardy group (Fig. 11), estimates of pairwise F_ST_ (Fig. 12), shifts in site frequency spectrum (Tajima’s D) in the Hardy (Fig. 13) and Not Hardy group (Fig. 14), scaled diversity (π_Hardy_/π_Not Hardy_) in the hardy group relative to the not hardy group (Fig. 15), and a case-control genome-wide association study (GWAS) for hardy vs not hardy (Fig. 16) are also shown.

Estimates of the local pairwise LD in the candidate region of chromosome 1 are shown for the hardy and not hardy groups in figures 17 and 18, respectively, with close-ups of the 29.00 – 29.05 Mb region in figures 19 and 20, respectively.


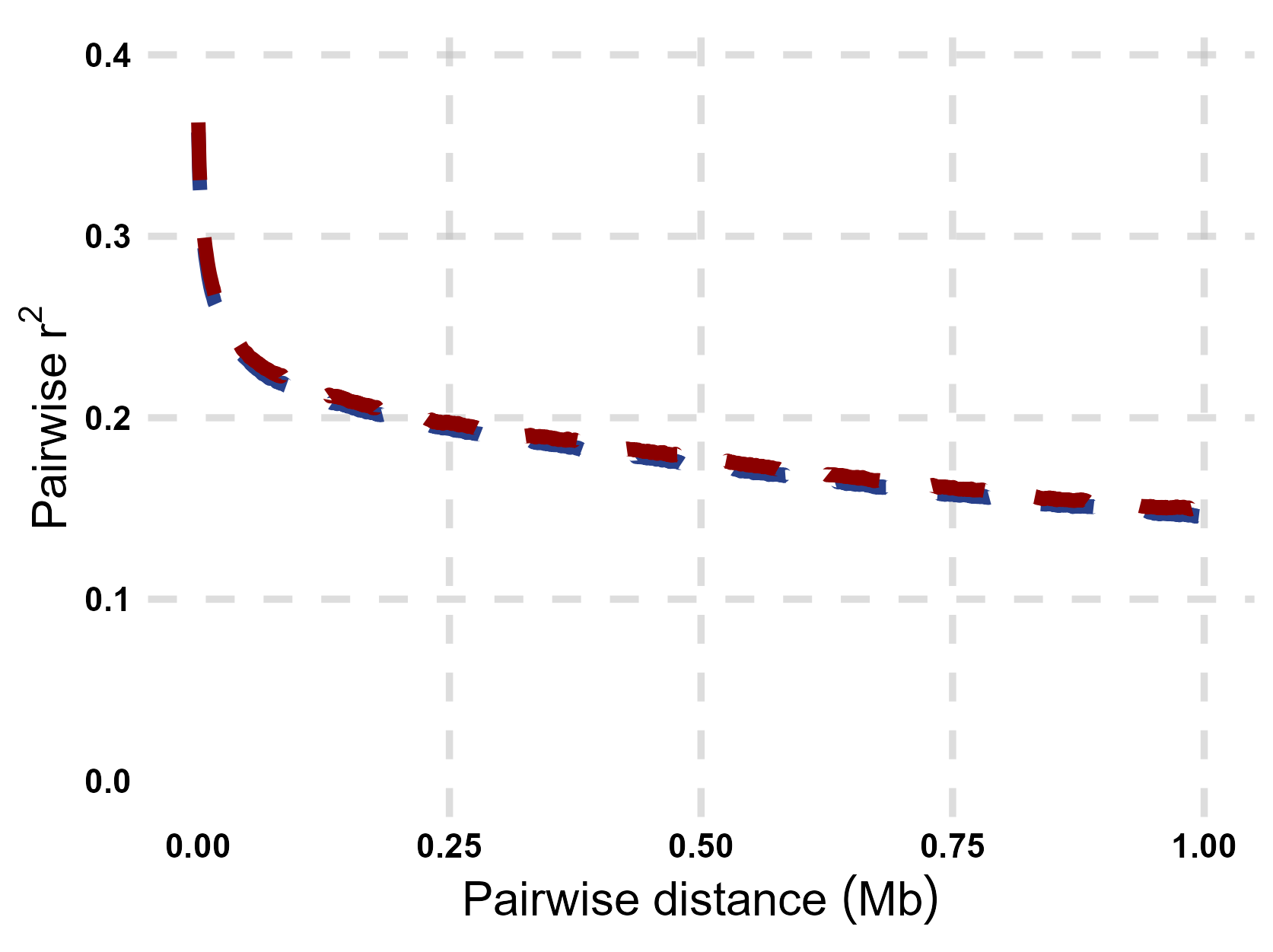


Fig. 1: Average pairwise R^2^ calculated in bins of 1 kb for the hardy and the not hardy groups separately, excluding ‘Släthultsäpple’, ‘Leckö Astrakan’, and ‘Noors Glasäpple’. The hardy group is indicated by a blue dashed line and the no hardy group is indicated by a red dashed line.


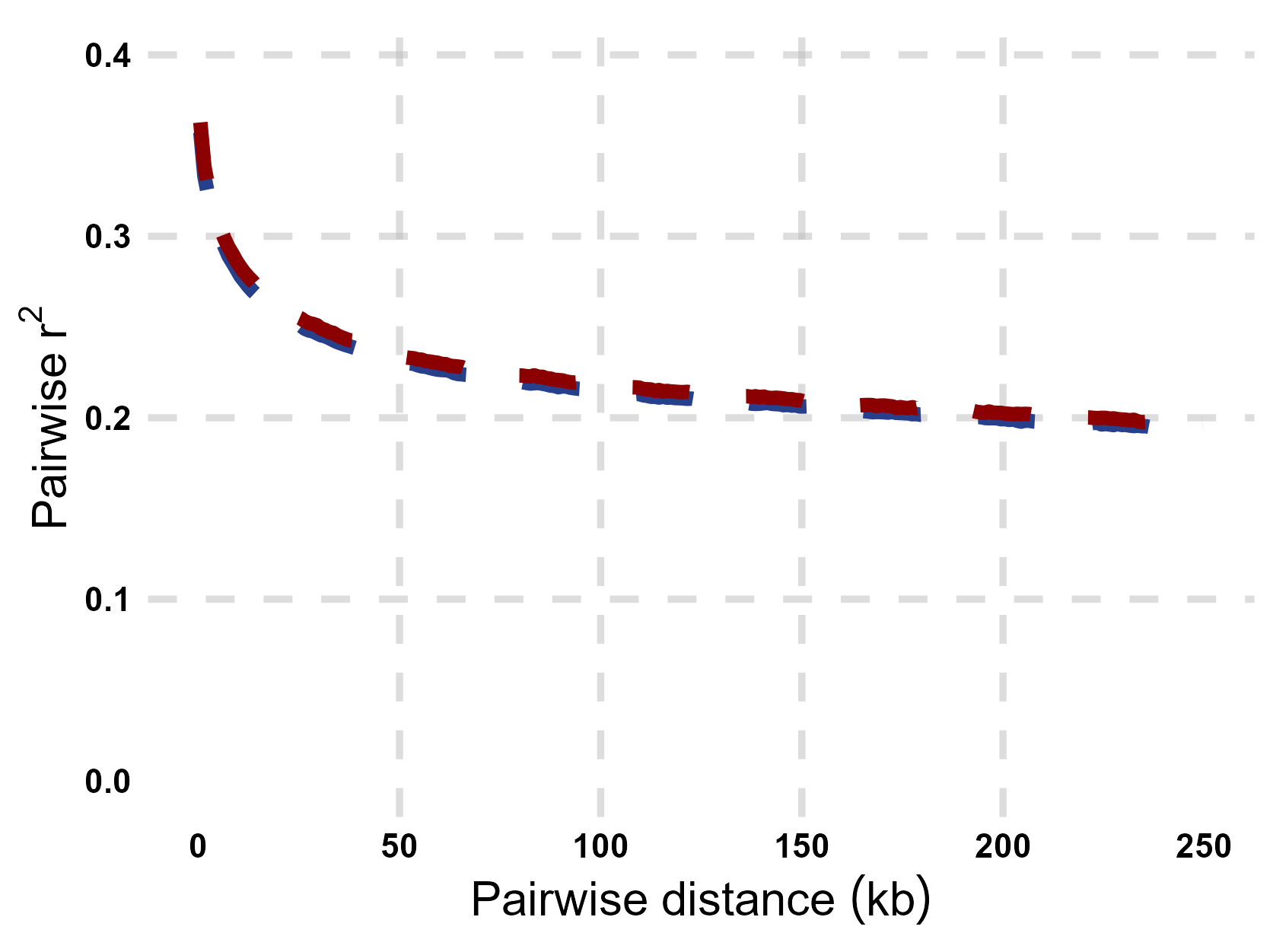


Fig. 2: Average pairwise R^2^ calculated in bins of 1 kb for the hardy and the not hardy groups separately, excluding ‘Släthultsäpple’, ‘Leckö Astrakan’, and ‘Noors Glasäpple’. The hardy group is indicated by a blue dashed line and the no hardy group is indicated by a red dashed line.


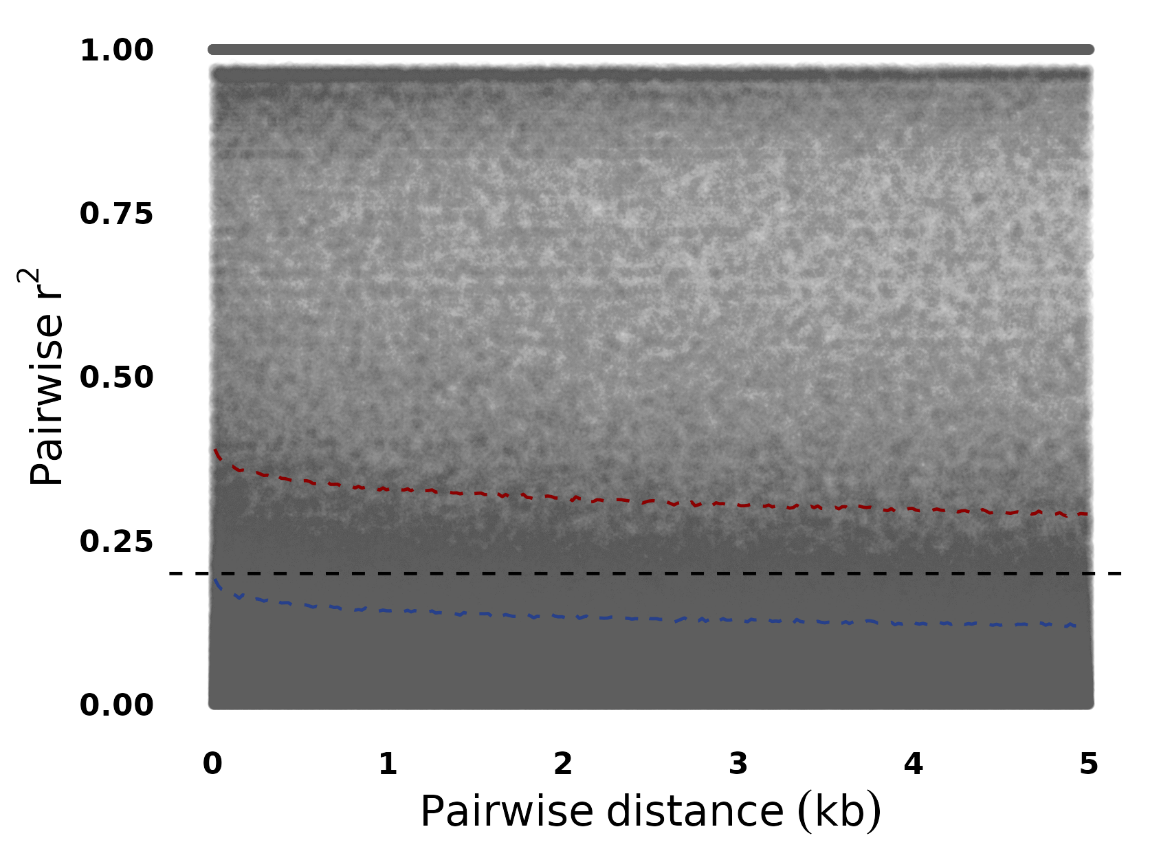


Fig. 3: Pairwise R^2^ calculated in bins of 20 bp for the entire data set up to 5 kb. The average R^2^ is indicated by a red dashed line and the median R^2^ is indicated by a blue dashed line. R^2^ 0.2 is indicated by a black dashed line.


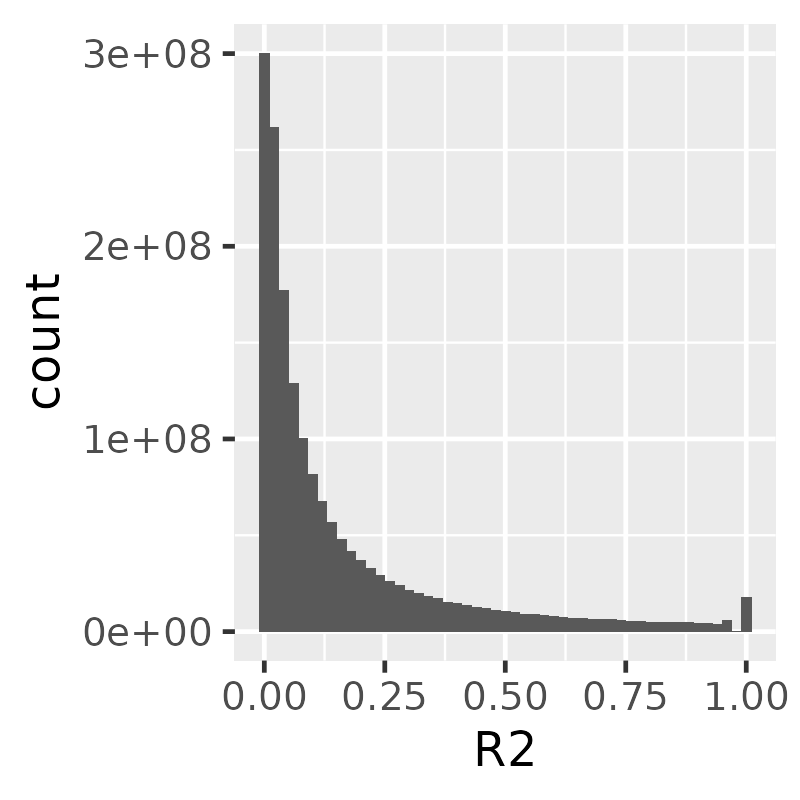


Fig. 4: Distribution of pairwise R^2^ values for pairs of loci up to 1 Mb apart.


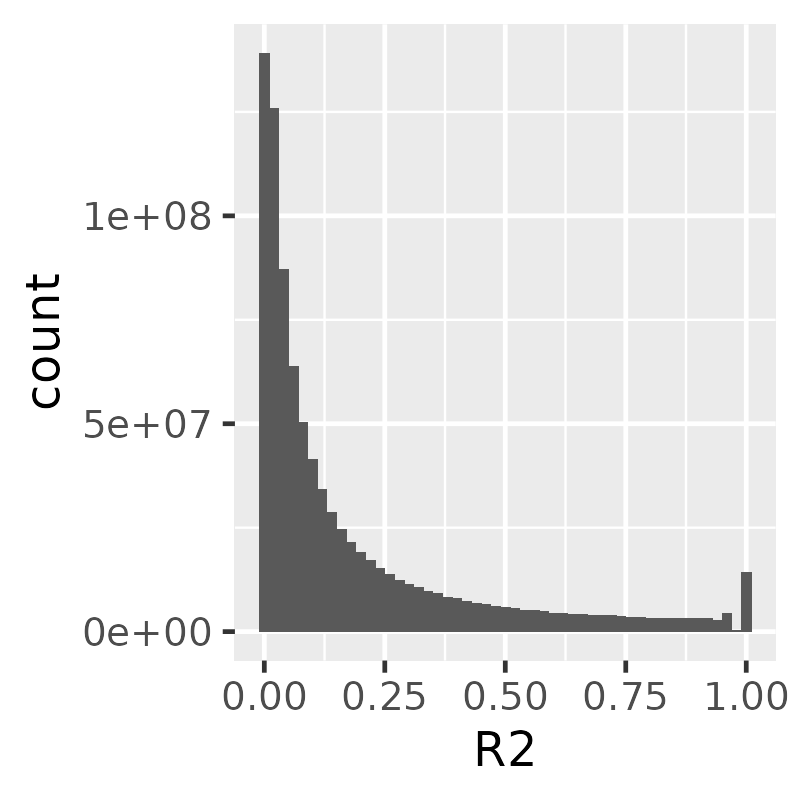


Fig. 5: Distribution of pairwise R^2^ values for pairs of loci up to 500 kb apart.


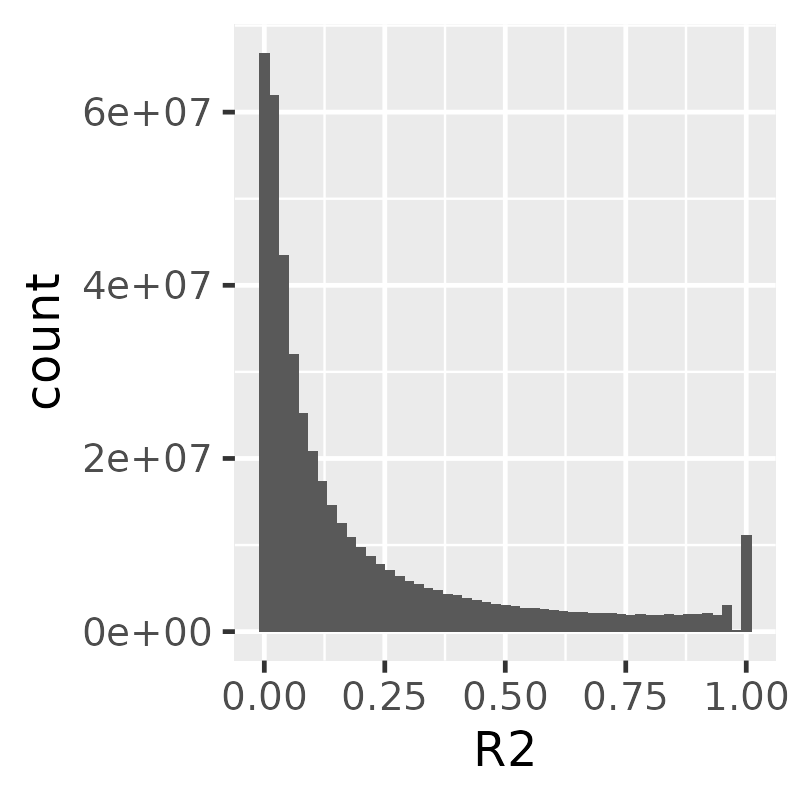


Fig. 6: Distribution of pairwise R^2^ values for pairs of loci up to 250 kb apart.


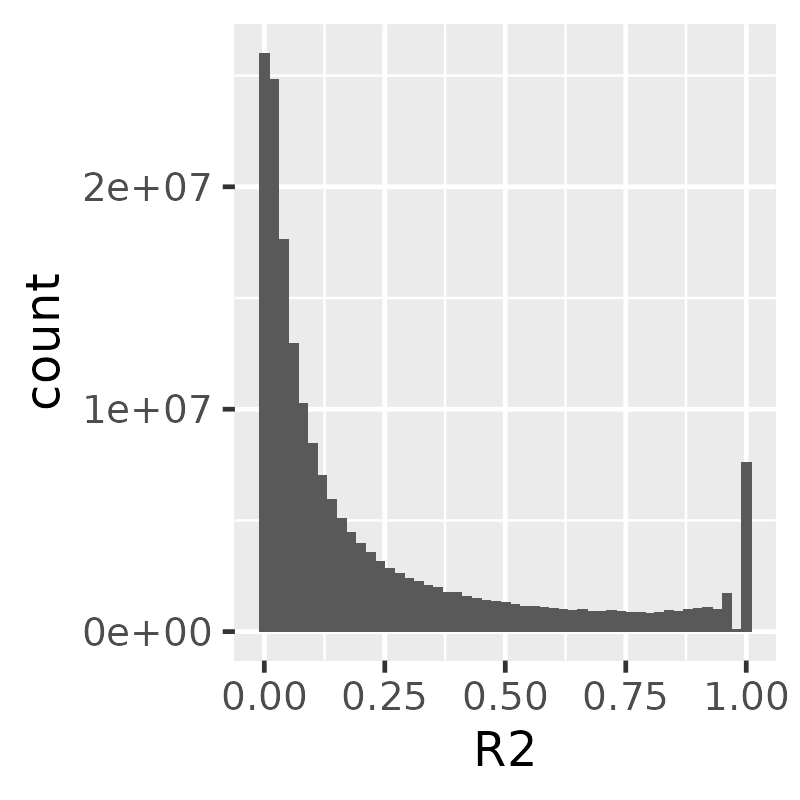


Fig. 7: Distribution of pairwise R^2^ values for pairs of loci up to 100 kb apart.


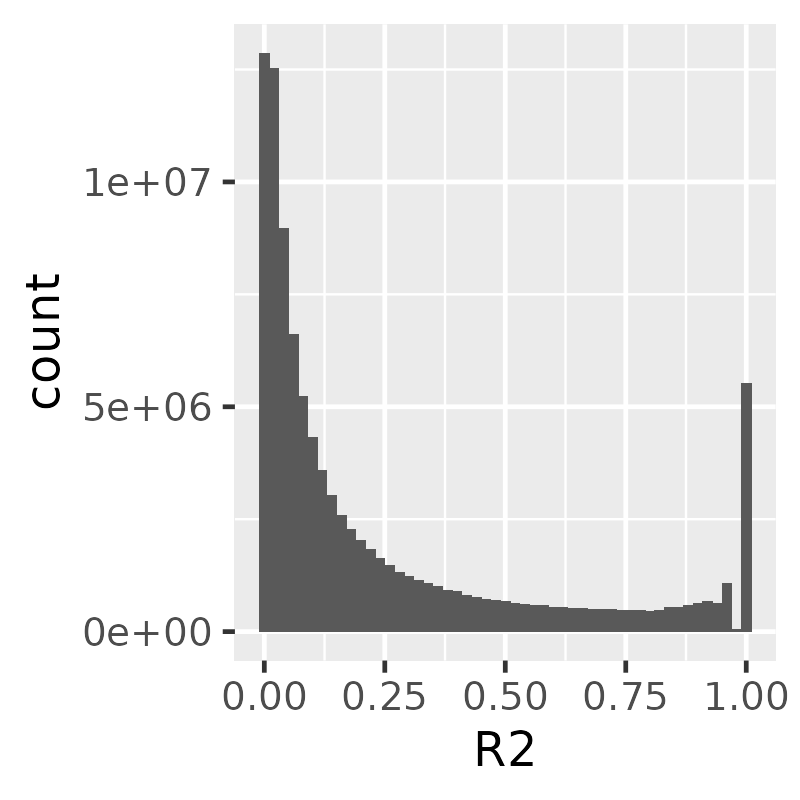


Fig. 8: Distribution of pairwise R^2^ values for pairs of loci up to 50 kb apart.


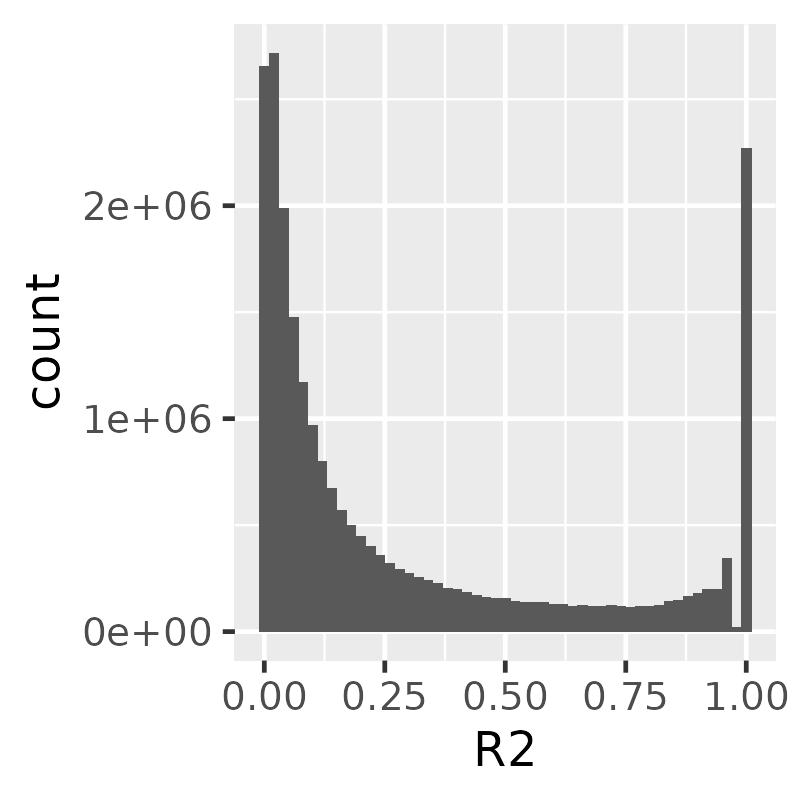


Fig. 9: Distribution of pairwise R^2^ values for pairs of loci up to 10 kb apart.


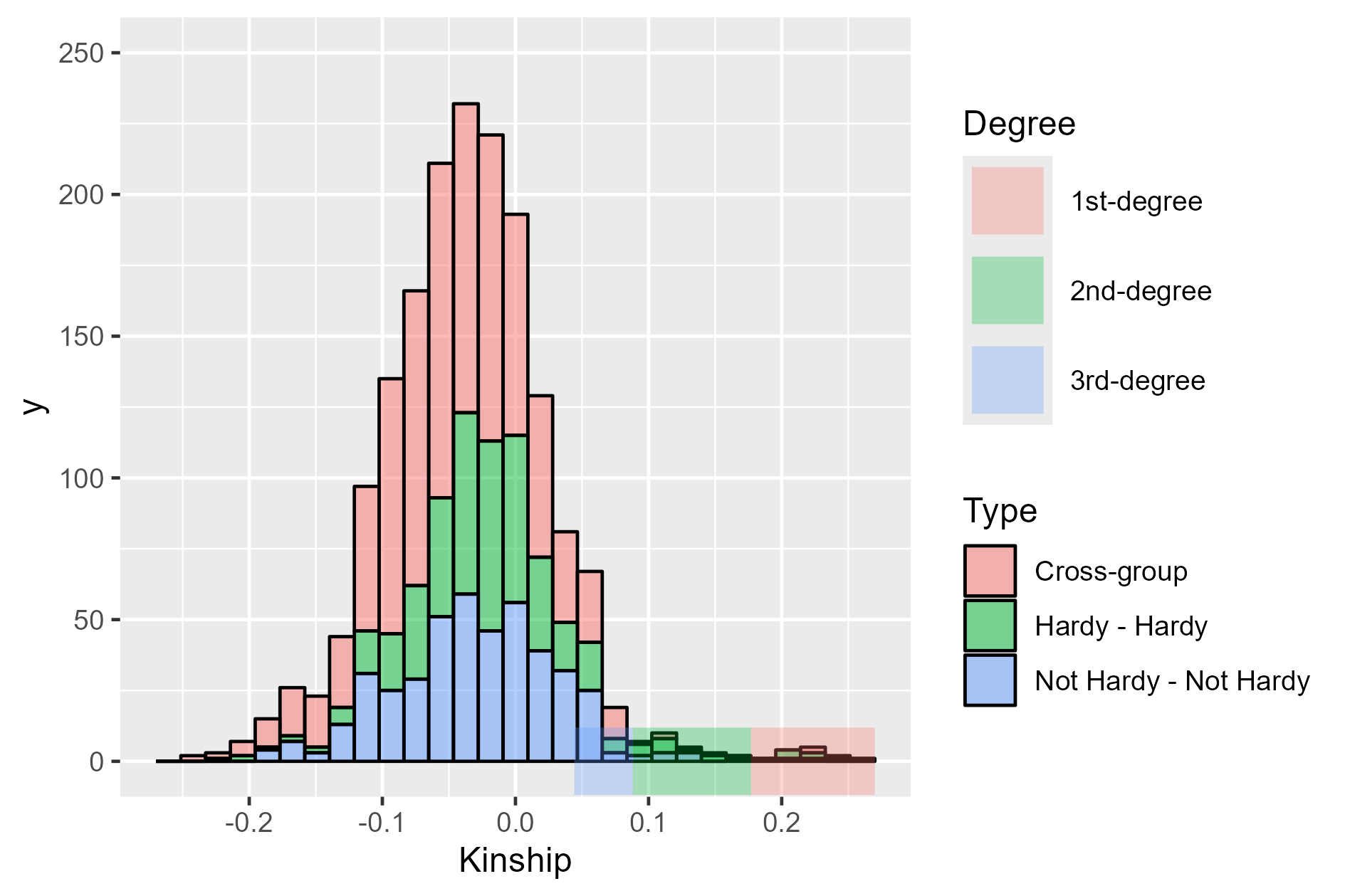


Fig. 10: Distribution of inferred pair-wise kinship coefficients, group by pairs within the two respective groups and pairs between groups. Negative values indicate an unrelated relationship, and the corresponding degrees of close relationships are indicated by transparent bars along the x-axis.


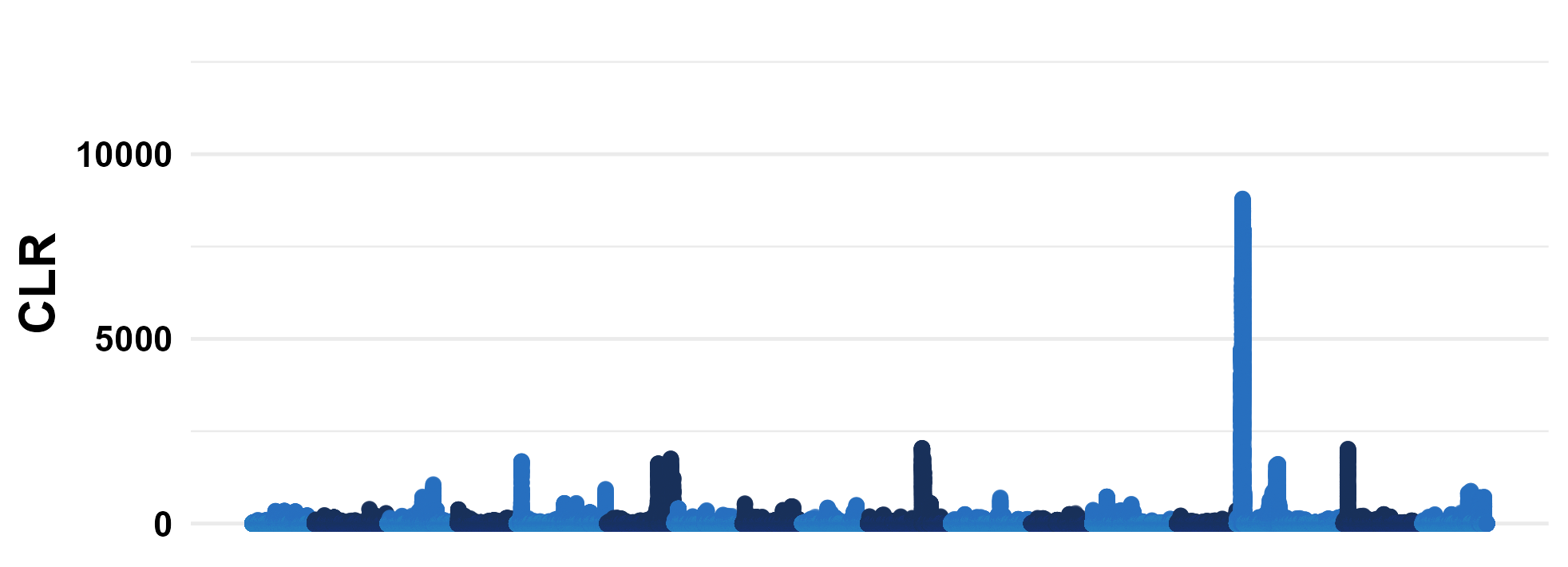


Fig. 11: Genome-wide results from composite likelihood ratio (CLR) tests for selective sweeps in the not hardy group.


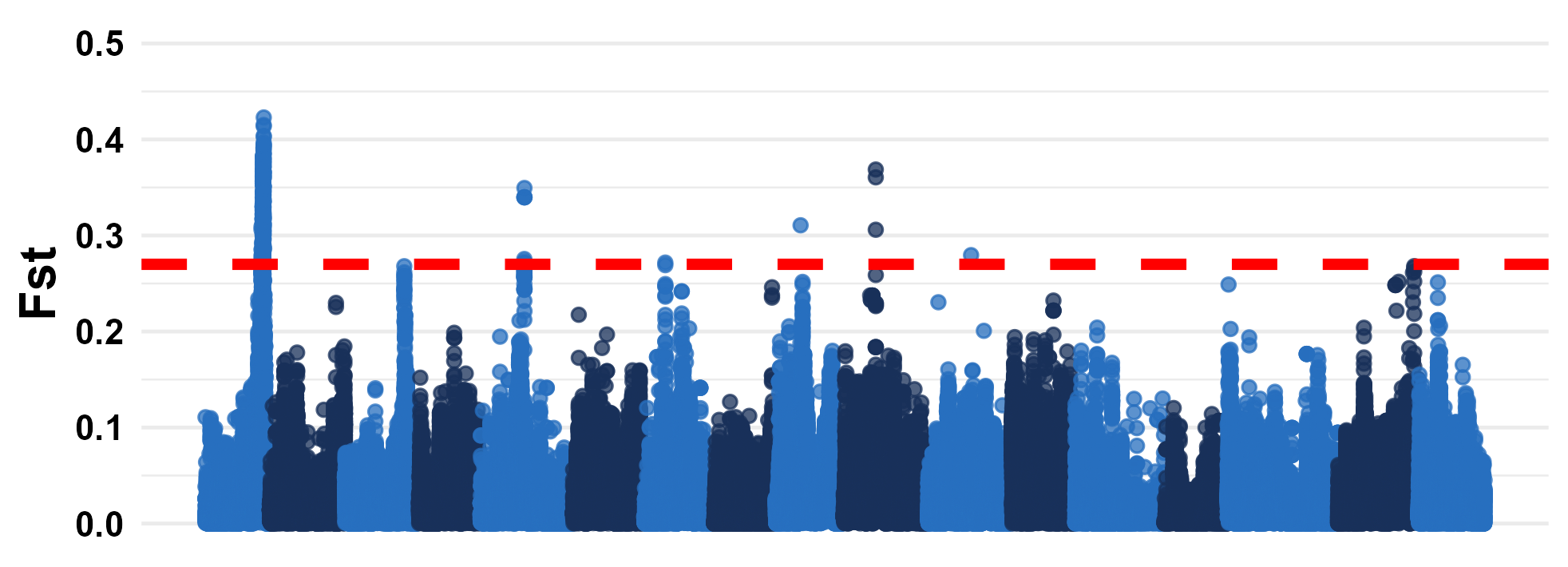


Fig. 12: Genome-wide weighted pair-wise F_ST_ between hardy and not hardy group.


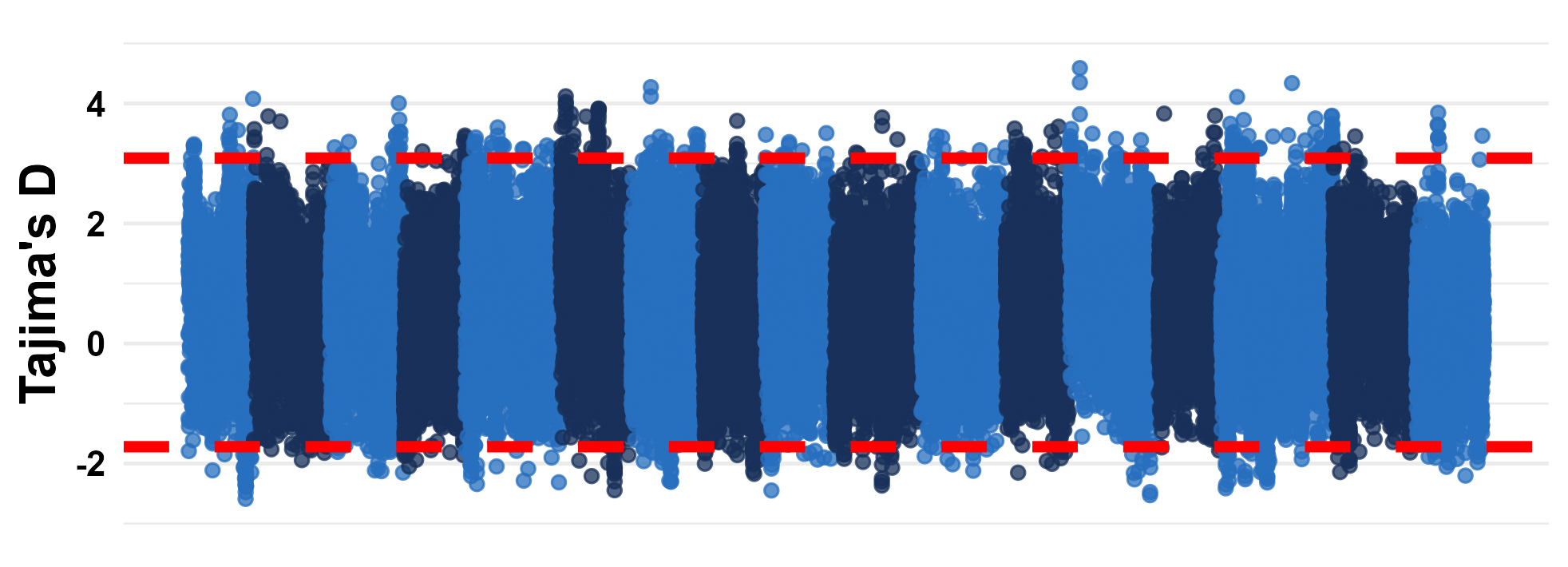


Fig. 13: Genome-wide Tajima’s D in the hardy group. Red dashed lines indicate two-sided thresholds for 1% outliers.


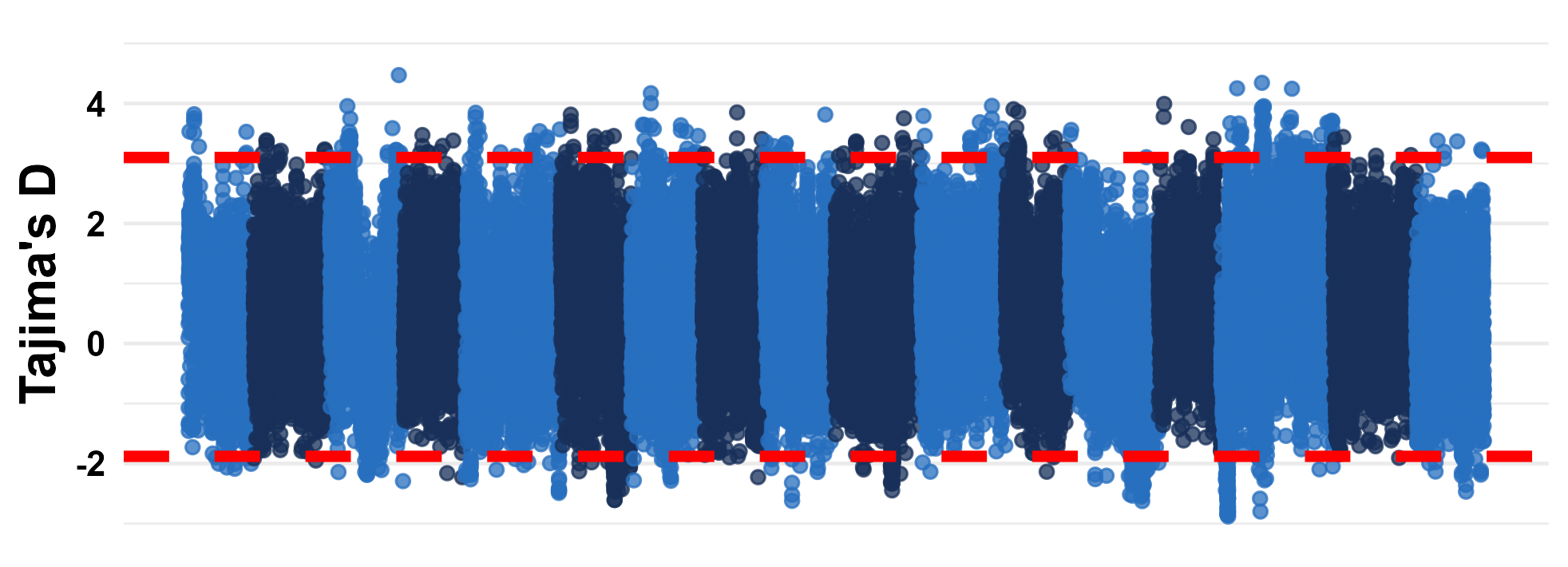


Fig. 14: Genome-wide Tajima’s D in the not hardy group. Red dashed lines indicate two-sided thresholds for 1% outliers.


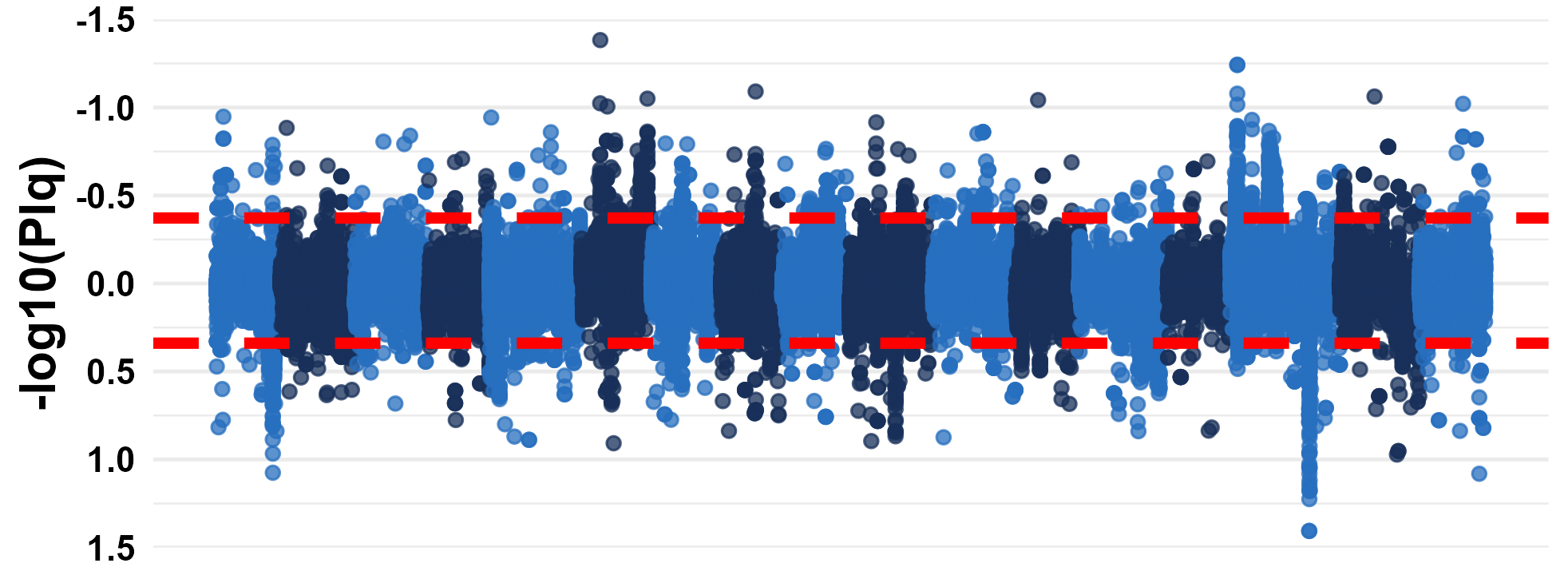


Fig. 15: Genome-wide scaled diversity in the hardy group relative to the not hardy group. Red dashed lines indicate two-sided thresholds for 1% outliers.


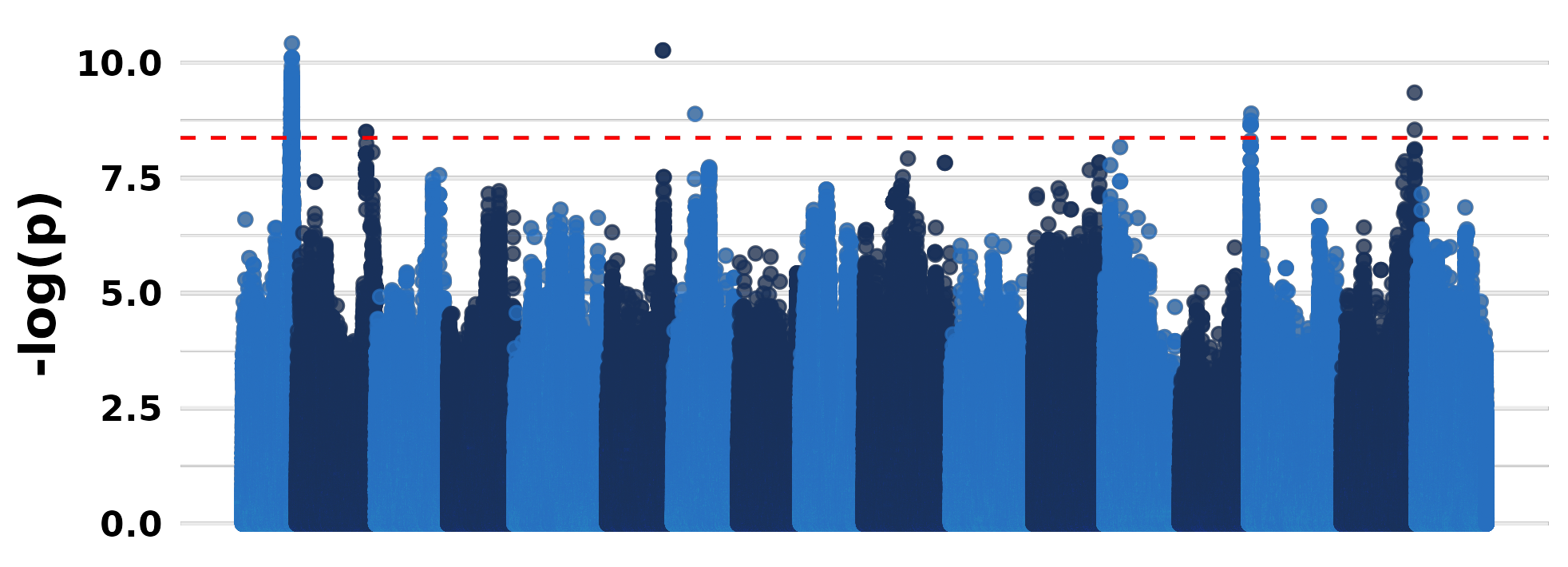


Fig. 16: Case-control GWAS with red dashed line indicating the threshold for p<0.05 following Bonferonni correction for false discoveries.


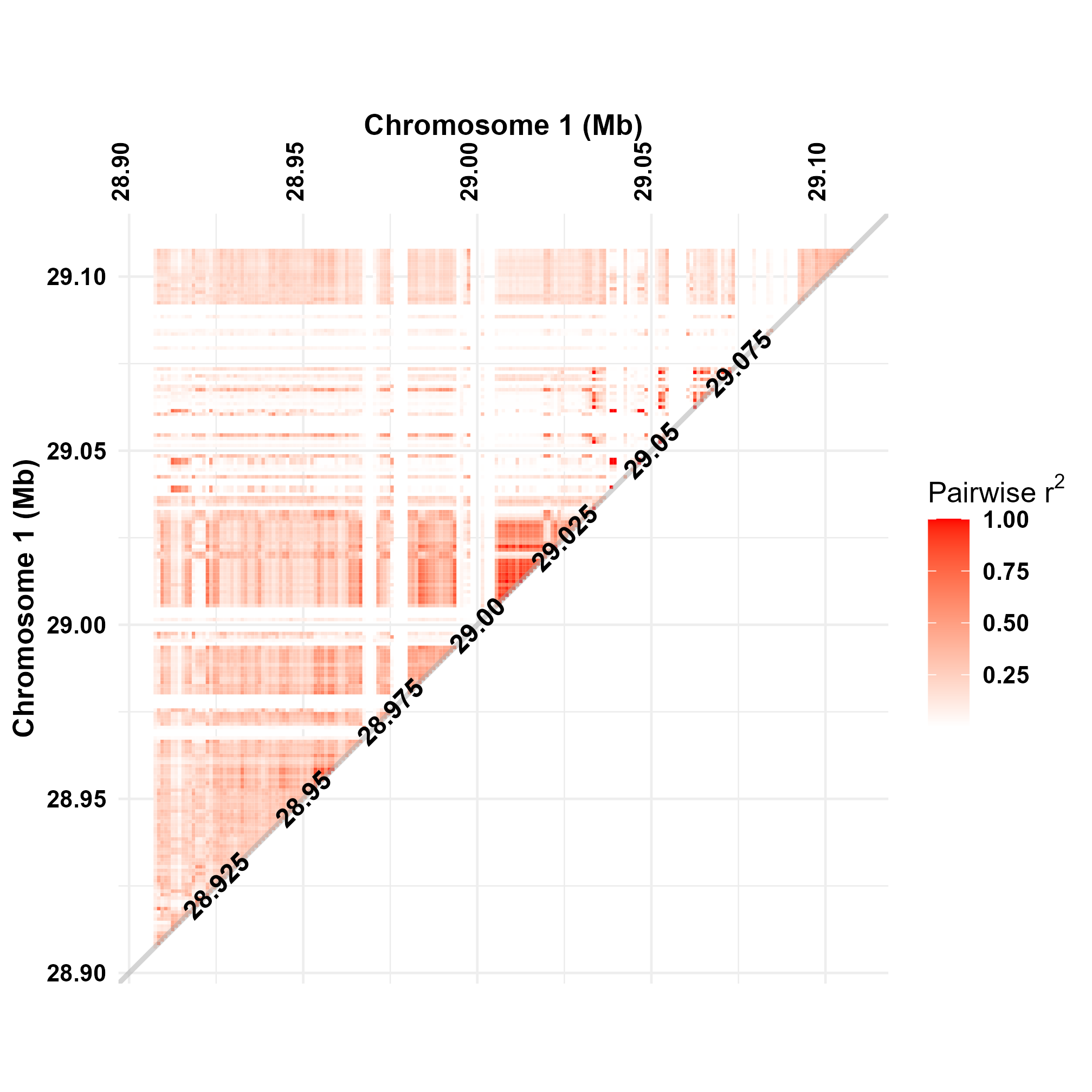


Fig. 17 LD heatmap of pairwise r^2^ in the hardy group between 28,907,237 and 29,107,237 bp of chromosome 1, plotted in bins of 1 kb.


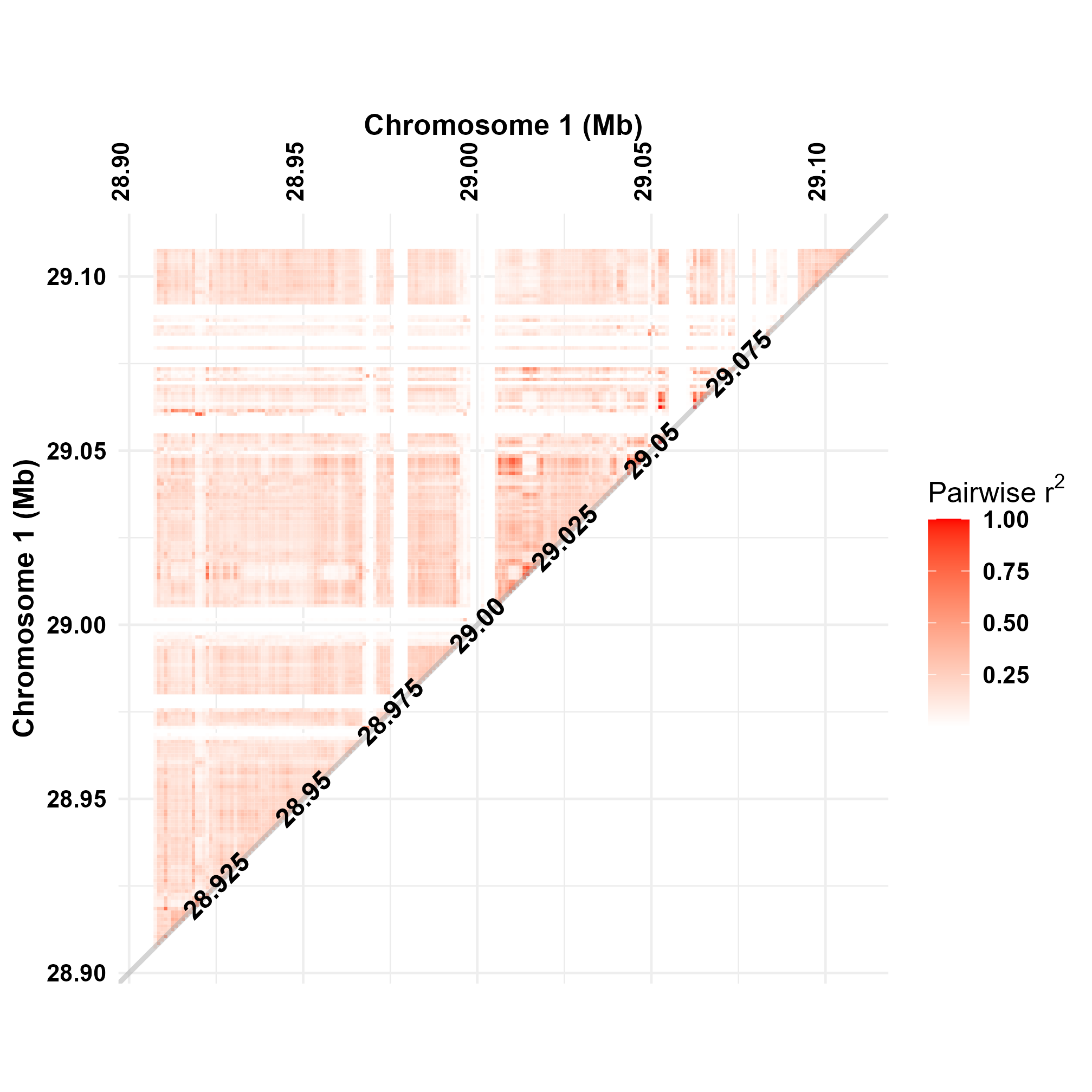


Fig. 18 LD heatmap of pairwise r^2^ in the not hardy group between 28,907,237 and 29,107,237 bp of chromosome 1, plotted in bins of 1 kb.


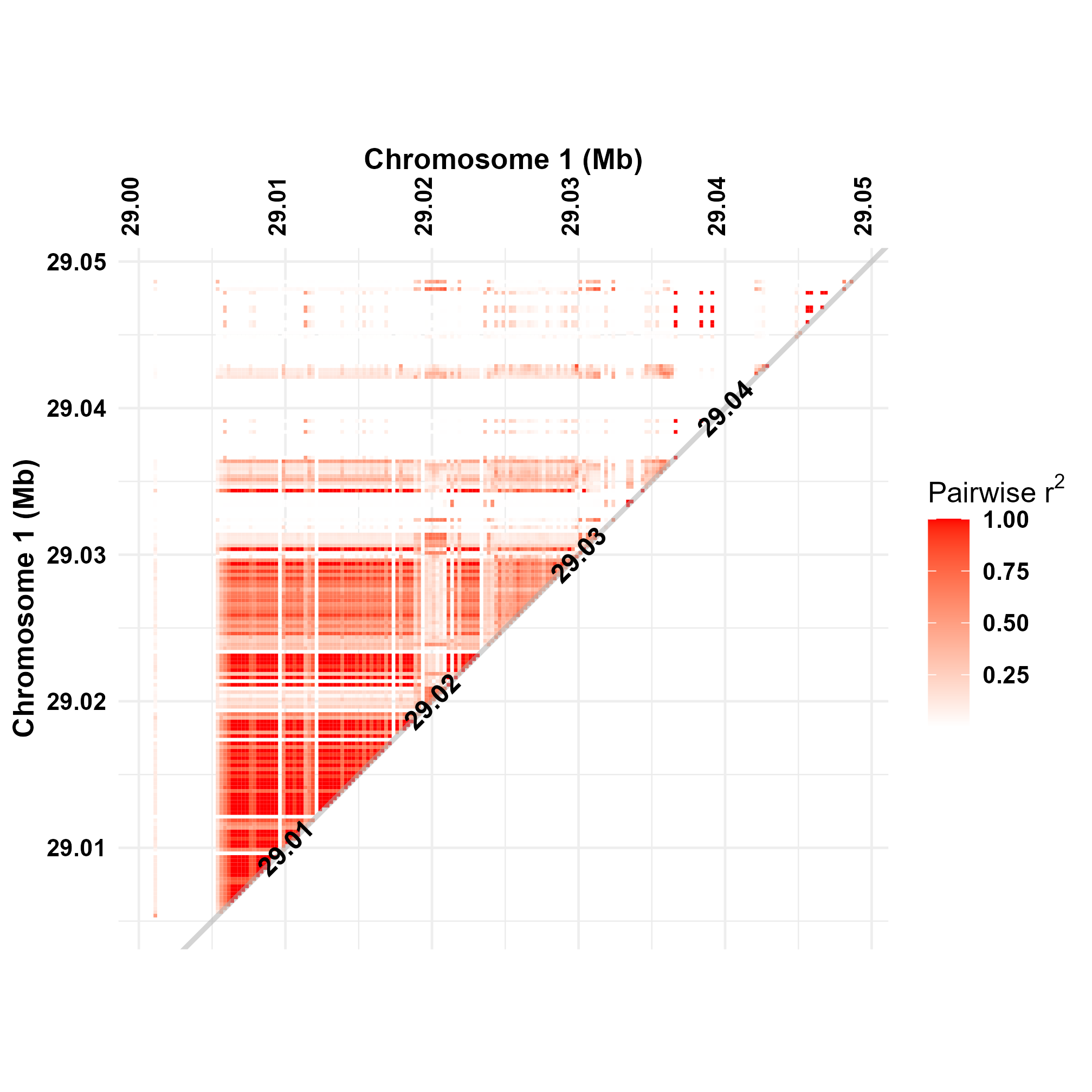


Fig. 19 LD heatmap of pairwise r^2^ in the hardy group between 29.00 and 29.05 MB, plotted in bins of 250 bp.


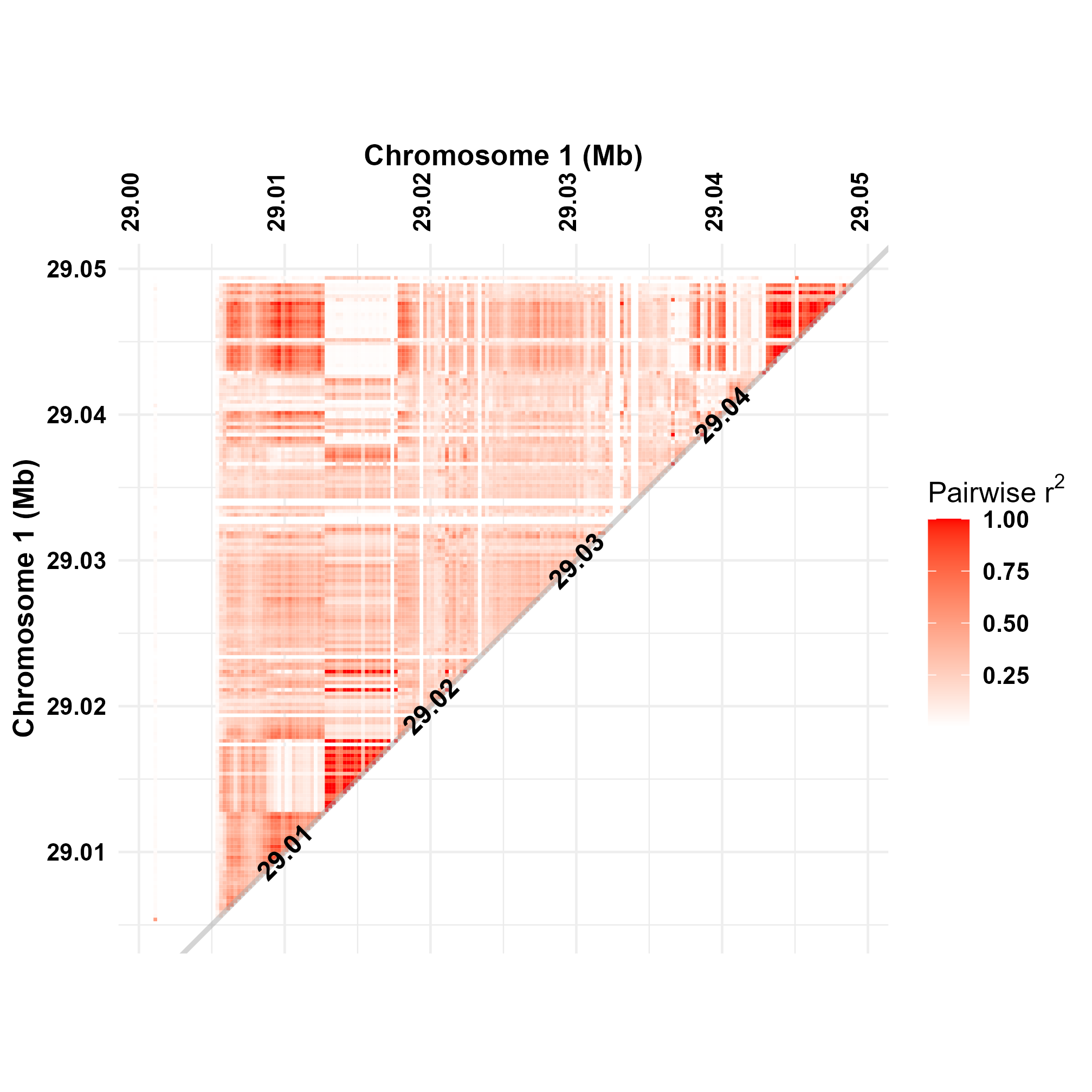


Fig. 20 LD heatmap of pairwise r^2^ in the not hardy group between 29.00 and 29.05 MB, plotted in bins of 250 bp.
